# Characterizing and correcting immune dysfunction in non-tuberculous mycobacterial disease

**DOI:** 10.3389/fimmu.2022.1047781

**Published:** 2022-11-10

**Authors:** Champa N. Ratnatunga, Katie Tungatt, Carla Proietti, Sam Halstrom, Michael R. Holt, Viviana P. Lutzky, Patricia Price, Denise L. Doolan, Scott C. Bell, Matt A. Field, Andreas Kupz, Rachel M. Thomson, John J. Miles

**Affiliations:** ^1^ Australian Institute of Tropical Health and Medicine, James Cook University, Cairns, QLD, Australia; ^2^ School of Medicine, University of Queensland, Brisbane, QLD, Australia; ^3^ Queensland Institute of Medical Research (QIMR) Berghofer, Brisbane, QLD, Australia; ^4^ Faculty of Medicine, University of Peradeniya, Kandy, Sri Lanka; ^5^ Centre for Molecular Therapeutics, James Cook University, Cairns, QLD, Australia; ^6^ Curtin Medical School, Curtin University, Perth, WA, Australia; ^7^ Thoracic Medicine, The Prince Charles Hospital, Brisbane, QLD, Australia; ^8^ Gallipoli Medical Research Institute, Greenslopes Private Hospital Foundation, Brisbane, QLD, Australia; ^9^ Centre for Tropical Bioinformatics and Molecular Biology, James Cook University, Cairns, QLD, Australia; ^10^ Division of Infection and Immunity, University Hospital Wales, Cardiff University School of Medicine, Cardiff, Wales, United Kingdom; ^11^ Systems Immunity Research Institute, Cardiff University, Cardiff, Wales, United Kingdom

**Keywords:** non-tuberculous mycobacteria, mycobacterial immunity, immune cell dysfunction, high-dimensional immunoprofiling, immune modulation

## Abstract

Non-tuberculous mycobacterial pulmonary disease (NTM-PD) is a chronic, progressive, and growing worldwide health burden associated with mounting morbidity, mortality, and economic costs. Improvements in NTM-PD management are urgently needed, which requires a better understanding of fundamental immunopathology. Here, we examine temporal dynamics of the immune compartment during NTM-PD caused by *Mycobacterium avium* complex (MAC) and *Mycobactereoides abscessus* complex (MABS). We show that active MAC infection is characterized by elevated T cell immunoglobulin and mucin-domain containing-3 expression across multiple T cell subsets. In contrast, active MABS infection was characterized by increased expression of cytotoxic T-lymphocyte-associated protein 4. Patients who failed therapy closely mirrored the healthy individual immune phenotype, with circulating immune network appearing to ‘ignore’ infection in the lung. Interestingly, immune biosignatures were identified that could inform disease stage and infecting species with high accuracy. Additionally, programmed cell death protein 1 blockade rescued antigen-specific IFN-γ secretion in all disease stages except persistent infection, suggesting the potential to redeploy checkpoint blockade inhibitors for NTM-PD. Collectively, our results provide new insight into species-specific ‘immune chatter’ occurring during NTM-PD and provide new targets, processes and pathways for diagnostics, prognostics, and treatments needed for this emerging and difficult to treat disease.

## Introduction

Non-tuberculous mycobacterial pulmonary disease (NTM-PD) is a rising global health problem. Epidemiology has shown marked increases in NTM-PD prevalence globally over the last two decades (1–7). *Mycobacterium avium* complex (MAC) and *Mycobactereoides abscessus* complex (MABS) account for approximately 80% of non-tuberculous (NTM) pathology (8).

The causative factors underlying NTM-PD are not fully understood. Current data suggest that patient, environmental and bacteriological factors interact to create a ‘susceptible host’ (9). NTM-PD infection is typically observed in adult patients with impaired lung function, including chronic obstructive pulmonary disease (COPD), bronchiectasis, and cystic fibrosis (10, 11), systemic immune compromise, including TNF antagonist therapy, cytotoxic therapy and steroid therapy (12) and in immune-competent individuals with specific anatomical and physiological characteristics. For example, elderly, white, females with low body mass index (BMI) with predisposition to scoliosis, pectus excavatum and mitral valve prolapse, a condition termed Lady Windemere syndrome (13)

NTM-PD therapy is complicated, expensive, and prolonged, requiring 12-18 months of multi-drug regimens that are difficult to tolerate. Disease remission rates vary depending on infecting species, patient age and comorbidities and are 50% or lower (14). Recurrent or relapsing NTM infections are common in NTM-PD, occurring in 30-50% of MAC NTM-PD patients (15) and up to 55% of MABS NTM-PD patients (16). Many patients develop chronic infection despite treatment, while others succumb to the disease (14). The combined factors of prolonged therapy, complex antibiotic regimes, increasing microbial resistance, and increasing prevalence make NTM-PD a severe and expensive health care problem (14) with costs of $US12,200-$US20,000 per patient annually for MAC treatment (17) and up to $US20,000 per patient per month for MABS treatment (16). In addition, NTM-PD patients have almost six times higher secondary care requirements when compared to related illnesses (11). Furthermore, there are alarmingly high mortality rates in patients following NTM-PD diagnosis, with five-year mortality at 27%-35% (18). A further concern of this emerging disease burden is the discovery of person-to-person transmission (19).

Given the escalating human and economic cost of NTM-PD infection, new screening methods are required to rapidly identify persons at risk and advise on clinical management. Immune suppressive receptors, such as the checkpoint receptors, programmed cell death protein 1 (PD-1), cytotoxic T lymphocyte-associated protein 4 (CTLA-4) and T cell immunoglobulin and mucin domain-containing protein 3 (TIM-3), have attracted significant interest as screening tools for a wide range of human diseases. Likewise, tuberculosis (TB) patients have elevated PD-1 expression on antigen-specific T cells and blocking the PD-1 axis increases effector cell degranulation as well as IFN-γ production (20). PD-1 and TIM-3 were also elevated on antigen-specific CD8^+^ T cells during viral infection in the mouse, and pathway blocking reversed exhaustion (21).

The fundamental biology underlying immune checkpoint activity are being defined (22, 23), primarily in oncology. However, is little known on immune checkpoint expression and kinetics during NTM pathology (24). From a clinical perspective, the lack of effective, side-effect-free, short-course, and long-lasting treatments urge further mechanistic research in NTM-PD. Here, we examined the circulating immune compartment at different NTM-PD stages. We identified novel biomarkers of active disease and treatment response and determined an immunological basis underlying persistent NTM-PD. In addition, we successfully blocked the PD-1 axis on patient samples to increase effector cell efficacy against NTM suggesting the redeployment of immune checkpoint drugs for NTM-PD.

## Methods

### Study approval

The study was approved by the QIMR Berghofer (QIMRB) Human Research Ethics Committee (HREC) (#P1479), Greenslopes Private Hospital HREC (12/12 & 14/14) and JCU HREC (#H7010). All protocols were carried out in accordance with site guidelines and regulations. Informed consent was obtained from all participants, and the study was conducted under the Declaration of Helsinki guidelines.

### Patient characteristics

Middle-aged and elderly patients with MAC or MABS pulmonary disease were studied at three disease stages. Disease stages were defined based on the American Thoracic Society 2007 guidelines for NTM-PD diagnosis (25). Patients with active infection were defined as symptomatic individuals with microbiological and radiographic (high-resolution CT) evidence of pulmonary infection. Patients with active MAC infection and patients with active MABS infection who had no known immune compromise and were not on immune suppressive medication were recruited prior to starting antibiotic therapy. HIV infection was excluded in most patients. Patients who were in remission were defined by completed antibiotic therapy and culture-negative sputum for 12 consecutive months. Due to time constraints, only MAC patients who were in remission (MAC PostTx) were recruited. Patients with persistent infection were defined as those who remained sputum culture-positive despite treatment and included patients who had either MAC or MABS infection. Healthy individuals, age, and gender-matched to the patient cohorts were recruited from volunteers. Bronchiectasis patients with no prior history of NTM lung disease were recruited as a ‘within disease’ control group.

### Blood processing and banking

PBMCs were separated from venous blood by Ficoll-Paque PLUS (GE Health) density gradient method and were cryopreserved in R10 medium (RPMI-1640- (Gibco 21870-076), containing 10% heat-inactivated Foetal Bovine Serum (FBS), supplemented with 10% DMSO (Sigma-Aldrich).

### Flow cytometric phenotyping

We developed a 16-colour panel that discriminated T cell, B cell, NKT cell, NK cell and monocyte subsets to enumerate and quantitate the immune checkpoints markers CTLA-4, TIM-3, and PD-1 (**Supplemental Table 1**, Panel 1). A second 12-colour panel was developed for non-classical effector cell profiling, including mucosa-associated invariant T cell (MAIT) cells and γδ T cells (**Supplemental Table 2**, Panel 2). PBMC were thawed, counted and viability-stained cells were washed and labelled with CCR7 in 25µl 2% FBS for 30 min. Subsequently, all other stains were added as a master mix (to a final volume of 50µl) to panel 1 as well as panel 2 cells and incubated on ice for a further 30 min. Cells stained with panel 1 were next stained for intracellular CTLA-4 and FOXP3 using the FOXP3 fixation/permeabilization kit (Invitrogen). A 15-colour panel (**Supplemental Table 3**, Panel 3) which included seven cytokine and functional markers was optimized to evaluate the functional capacity of PBMCs. A 500X Cell Stimulation Cocktail (Invitrogen) containing 50 ng/mL of Phorbol myristate acetate (PMA) and 1 µg/mL of ionomycin was added at a final concentration of 1x to 1x10^6^ cells in R10 medium. Cytokine secretion was blocked using the 500X Protein Transport Inhibitor Cocktail (eBioscience) containing brefeldin and monensin at a final concentration of 1x. Unstimulated control wells determined baseline levels of cytokine secretion. Selected stains were added to cell culture media, including lineage markers CD3, CD4, CD8, CD56 and CD14 and degranulation marker CD107a. Following a 6 hr incubation, cells were washed, resuspended in PBS and stained with a viability dye, CCR7 and CD45RA stain in sequence on ice for 20 mins each. Cells were then washed and stained for intracellular cytokines using the Cytofix/Cytoperm Permeabilization kit (BD Biosciences).

### Antigen-specific recall

Antigen-specific recall was quantified by overnight stimulation with antigen and intracellular staining (ICS) utilizing an optimized 12 colour proliferation panel (**Supplemental Table 4**, Panel 4). As specific immunogenic epitopes for NTM have not been identified, CD40L (CD154), which has been used in the human TB setting (26), was used as a surrogate marker of antigen-specific T cell activation (27, 28). Therefore, proliferating CD40L^+^ CD4^+^ T cells and proliferating CD8^+^ T cells were quantified as antigen-specific T cells. Cells were stained with Violet Proliferation Dye 450 (VPD450 BD) (BD Biosciences). After seven days of incubation with antigen, cells were re-stimulated overnight with crude MAC antigen or PPD. A 500x Protein Transport Inhibitor Cocktail (eBioscience) was added after 4 hrs of incubation with antigen at a final concentration of 1x. On the following day, cells were transferred to a 96-well -plate and resuspended in PBS. Cells were then stained for viability and surface stains, and subsequently permeabilized with a Cytofix/Cytoperm kit (BD Biosciences). and stained for intracellular CD40L (28) and intracellular cytokines.

### Flow cytometric data acquisition and analysis

Samples were acquired on LSR-II 5-laser, 18 parameter Fortessa (BD Biosciences). Samples were acquired using FACSDiva software (BD Biosciences). Automated compensation using single stained compensation beads, experiment templates and application settings were used to maintain consistency between batch runs. Quality control measures included dead cell, doublet and flow rate anomaly exclusion, gating controls using relevant fluorescence-minus-one (FMO) controls and technical replicate-based run controls. Cell proliferation indexes were automatically calculated based on the proliferation model applied to each sample. Gating strategies used for each panel are shown in **Supplemental Figures 3**–**5**. Single, viable CD14^−^, CD16^−^, CD19^−^, CD3^+^ lymphocytes in each lineage (CD4^+^ or CD8^+^) were then analyzed using FlowJo version 10 (FlowJo LLC).

### Fluorescence-activated cell sorting, RNA extraction and gene quantification

CD4^+^and CD8^+^ T cells were sorted using fluorescence-activated cell sorting (FACS) for gene expression analysis as previously described (29). Briefly, un-thawed PBMC were rested for 1 hr in DNAse and 1.0-1.5x10^6^ viable cells were then counted, stained, and sorted into CD3^+^ CD4^+^ and CD3^+^ CD8^+^ T cell populations using a BD FACSAria III Sorter (BD Biosciences). Cells were collected into 400µl of R10 medium and stored on ice for RNA extraction. Frozen cell pellets were lysed in RNAzol RT (Astral Scientific) and centrifuged to separate total RNA. The aqueous phase was mixed with isopropanol (Sigma-Aldrich) and glycogen (Thermo Fisher Scientific) to precipitate RNA, and purified by ethanol washings (Sigma-Aldrich). RNA was quantified with a Nanodrop 2000. NanoString gene expression quantification was performed as previously described (30), according to the manufacturers instructions using RNA extracted from CD4^+^ and CD8^+^ sorted T cells. Approximately 100-150ng of RNA was used per sample.

### αCD3/αCD28 stimulated cellular proliferation

The proliferative capacity of T cells under non-specific αCD3/αCD28 antibodies stimulation was tested to evaluate if T cell proliferation was defective in patients with active or persistent lung infection with MAC. Prior to stimulation, cells were labelled with fluorescent proliferation dye (VPD450 BD) as per the manufacturer’s instructions. Cells were resuspended in R10 medium containing GlutaMAX-1 (100X Gibco, USA) 1x at 4x10^6^ cells/mL. 500µl of cell suspension was added to a 48-well plate. Purified NA/LE (no azide/low endotoxin) anti-human CD3 (clone HIT3a; 1.0mg/mL) was added to a final concentration of 1µg/ml to each well along with purified NA/LE anti-human CD28 (clone CD28. 2mL) to a final concentration of 5µg/mL as per optimized protocol. Culture plates were incubated at 37^°^C for 8 days. On day 7 of incubation, cells were re-stimulated with αCD3/αCD28 antibodies and incubated overnight with protein transport inhibitor and analyzed by flow cytometry.

### Antigen preparation

Antigen-specific responses were assessed by stimulating PBMCs with two forms of antigen. Crude antigen from five heat-killed cultures (pooled) of clinical MAC isolates obtained from the Queensland Mycobacterial Reference Laboratory. Protein content was quantified using the bicinchoninic colorimetric assay (BCA) (ThermoFisher). As this crude antigen extract likely contained immune-stimulating compounds including endotoxins, clinical-grade Purified Protein Derivative (PPD) (Sanofi Aventis) was used for validation. Tuberculin PPD was quantified using BCA. Both crude antigen and PPD concentrations were titrated, with and without IL-2 stimulation for protocol optimization.

### *In vitro* immune modulation

2x10^6^ cells per well were stained with VPD450 as per protocol described and plated in 48 well cell culture plates (Corning). Cells were stimulated with crude antigen at 10µg/mL and PPD at 40µg/mL Immune augmentation using αPD-1 was performed using PPD stimulation. Wells were treated with 10µg/mL of αPD-1 or 10µg/mL of the isotype control antibody Ultra-LEAF™ Purified Human IgG4 Isotype Control (Biolegend). Clinical-grade Nivolumab was graciously provided by Rajiv Khanna (QIMR Berghofer MRI, Australia). Control wells with no antigen established baseline levels of cytokine secretion. Culture plates were incubated in humidified 5% CO2 incubator at 37^0^C for 7 days. On day 7, 150µl of culture supernatant was harvested from all wells and stored at -80^0^C for CBA. Crude antigen and PPD were added, and culture plates were incubated for 4 hrs for antigen uptake. Protein transport inhibitor was then added, and cells were incubated overnight. The antigen recall response was quantified by intracellular staining and flow cytometry.

### Cytometric bead array

CBA (BD Biosciences) was next performed on supernatants from 7-day PPD stimulated cultures. The cytokines IFN-γ, TNF, IL-8, IL-10, IL-2, and IL-1β were quantified. Here, 20µl of supernatant was diluted 1:2 in 96 well plates, CBAs performed according to manufactures’ instructions. Samples were acquired on the BD LSRFortessa II Cell Analyzer (BD Biosciences). Standard curves were calculated for each analyte and analyte concentrations for referencing. If a standard curve failed to provide a good R^2^ and fit, median fluorescence intensity (MFI) of the analyte was reported.

### Statistical analysis

Univariate comparisons of cohort characteristics used Dunnetts T3 *post hoc* p >0.05 (**Table 1**) or ANOVA with Tukeys *post hoc* for BMI. Flow cytometric data were analysed using SPSS 25 v25 (IBM) and Prism v7 (GraphPad Software). Comparisons of cell percentage or MFI across multiple cohorts was performed using the Kruskal Wallis (KW) test with Dunns *post hoc* comparison of means if the KW test was significant. Comparisons of cohorts were performed using the Mann-Whitney U test. Significant values of p ≤ 0. 05 are reported. Boolean gating applied to single marker positive gates were formatted for use with Pestle (drmr.com/pestle.zip) and subsequently analyzed for patterns of marker expression using the Simple Presentation of Incredibly Complex Evaluations (SPICE) tool (niaid.github.io/spice, Beckman Coulter) (31). Data were analyzed to compare overall phenotype and polyfunctionality patterns per cell subset between cohorts and specific combinations of markers in specific cell subsets between patient and healthy controls. FCS files were uploaded to Cytobank (premium.cytobank.org, Beckman Coulter), visualized with the Visual Stochastic Neighbor Embedding (viSNE) tool and quantified with the cluster identification, characterization, and regression (CITRUS) tool (32). For antigen stimulation experiments, KW was performed with Dunns multiple comparisons to produce fold change values. All cell percentages were divided by the values of unstimulated controls.

**Table 1:** Demographic and patient characteristics.

	Active MAC	Active MABS	PostTx MAC	Persist inf.	BronchC	HC
Sample (n)	23	8	22	9	11	23
Mean age –Yrs (SD)	67.17 (6.43)	75.0 (8.19)	66.91 (9.99)	67.11 (8.95)	74.81 (8.91)	64.40 (8.84)
Female: Male	15:8	6:2	21:1	9:0	8:3	15:7
Mean BMI (kg/m^2^) (n)	22.83 (8)	19.7 (2)	22.11(13)	20.8 (5)		26.47 (11)
Species (n)	Ma^a^ (4)		Ma^a^ (3)	Ma^a^ (2)		
	Mi^b^ (19)		Mi^b^ (15)	Mi^b^ (4)		
	Other MAC^c^ *spp* (0)		Other MAC^c^ *spp* (4)	Mt^d^ (1)		
				MABS^e^ (2)		
History of prior infection	1	0	1			
Disease type						
NB	18	8	20	6		
NB and Cavitatory	3	0	2	2		
Cavitatory	2	0		1		
Treatment outcome						
Remission	13	3				
Persistence	5	2				
Remission with subsequent recurrence/relapse	2	2				
Other^f^	3	1				
Post treatment sampling						
<1year			8			
1-2years			5			
2-5years			5			
>5years			4			
Comorbidity						
Malignancy	6	0	4	0		1
GORD	2	1	2	2		3

a Mycobacterium avium.

b Mycobacterium intracellulare.

c Mycobacterium avium complex.

d Mycobacterium triplex.

e Mycobacterium abscessus complex.

f Other, Untreated or lost to follow up.

NB, Nodular bronchiectasis.

COPD, Chronic obstructive pulmonary disease.

GORD, Gastro-oesophageal reflux disease.

### Gene expression data

Data were run through the quality control pipeline in nSolver (NanoString Technologies), and samples that failed quality control were omitted. The Log2 values of these normalized counts were exported and analysed using Gmine (cgenome.net/GMine/) tool (30) and the R statistical package. Multivariate principal component analysis (PCA) and redundancy analysis (RDA) was used to identify potential underlying patterns (latent variables) that showed broad differences between patient and control cohorts in gene expression. Biomarkers that differentiated cohorts were determined using unpaired *t*-tests with p values adjusted for multiple comparisons at false discovery rate (FDR) of p<0.05. Multiple cohorts were compared by ANOVA tests again with FDR adjustment. *Post hoc* analysis of significant genes for mean separation was performed using Tukeys test. Gene expression signatures associated with active infection were determined by splitting the data set into a training set (randomly selected on 70% of all data) and a validation set (randomly selected on 30% of the remaining data). Random forest models were used on the training set to rank each gene based on importance in differentiating samples into the defined cohorts (active NTM-PD versus control BronchC or HC). The model was then tested on the independent validation data set. This process was repeated five times. Variable importance (mean decrease in model accuracy) for each gene was used as a measure for its predictive importance in discriminating active from control. Within each iteration, models based on different top-ranked genes were tested for their performance on the validation dataset by calculating the model’s sensitivity, specificity, and accuracy. Assuming genes that appeared in potential models more frequently were more important as classifiers, a final model was built. This model was then tested on the whole data set using the Leave One Out Classifier (LOOC) method, and model accuracy was calculated. A second random forest modelling on NanoString gene expression data for 105 TCR associated genes was performed. Data was input to the R package randomForest v4.6-14 to build the model. Forecasting was developed using 70% of data for training and 30% for validation using the R package caret v6.0.90. Annotation was performed using a fold change of cytokines over unstimulated samples along with the Log2 of these ratios.

## Results

### Patient demographics and clinical measures

**Figure 1:**
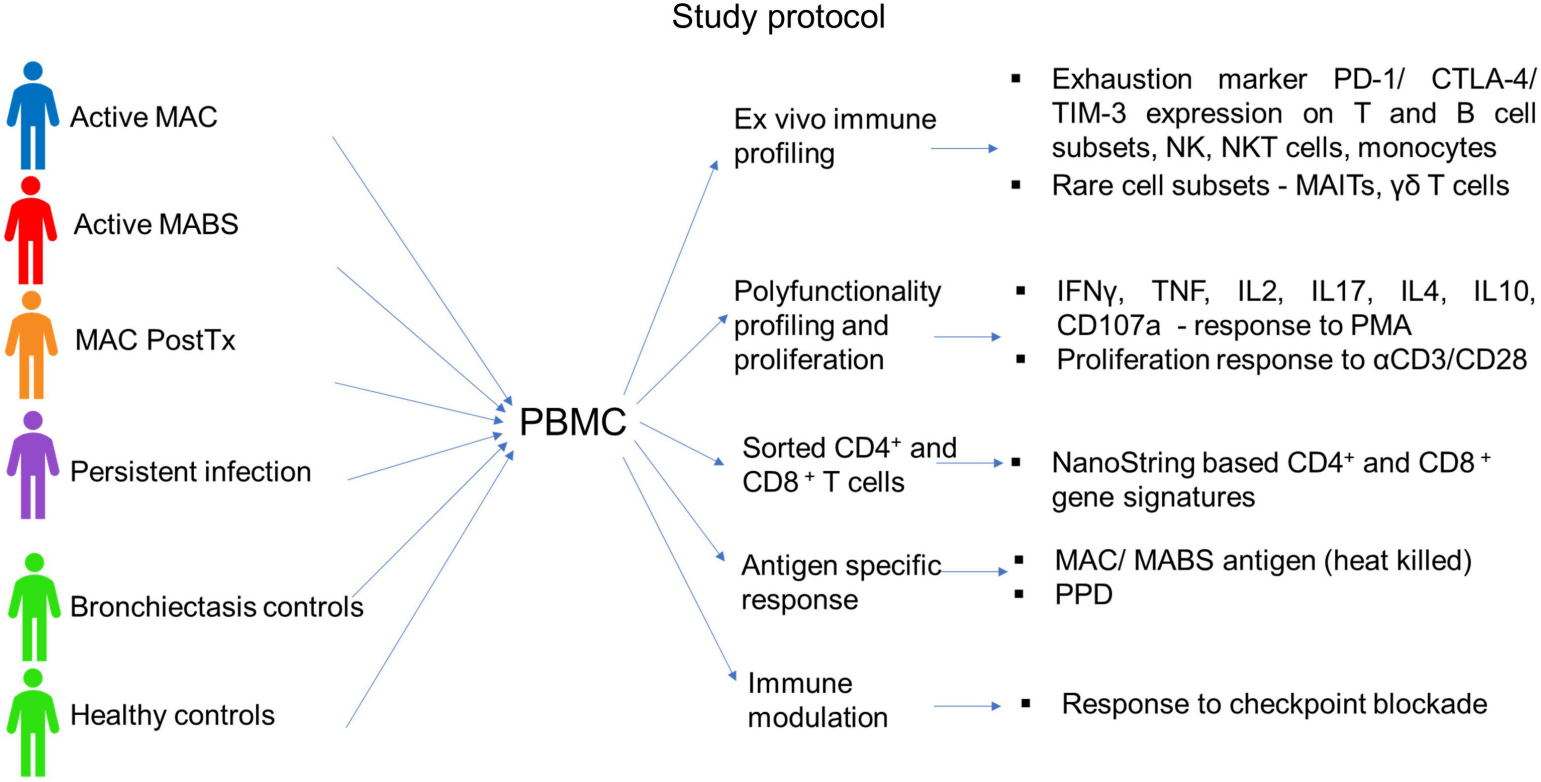
Study protocol. The following cohorts were included in the study: Active MAC (blue) – Patients with active pulmonary MAC infection prior to treatment. Active MABS (red) – Patients with active pulmonary MABS infection prior to treatment. PostTx MAC (orange) – patients with pulmonary MAC infection who had successfully completed the treatment course and were in disease remission (one month to >5 years post-treatment). Persistent infection (purple) – Patients who had persisting infection despite completed treatment courses. Bronchiectasis controls (green) – Matched controls diagnosed with bronchiectasis with no history of NTM pathology. Healthy controls (green) – Matched controls who had no underlying lung abnormalities, pathology, or history of NTM pathology.

A total of 96 subjects were included in the study (age range 64-75 years). Patients were predominantly female (76%) with no significant difference in age between cohorts (**Table 1**). Cohorts comprised patients with active MAC (active MAC) or MABS (active MABS) infection, MAC patients post-treatment who were in remission, (PostTx MAC) and patients with persistent infection despite treatment (Persist Infect), healthy controls with no history of NTM-PD (HC) and a ‘within disease’ control cohort of bronchiectasis patients with no history of NTM-PD (BronchC) (**Figure 1**).

BMI was significantly lower in the active MAC cohort (22.8 kg/m^2^) PostTx MAC cohort (22.1kg/m^2^), and the Persist Infect cohort (20.8kg/m^2^) compared with HC (26.5kg/m^2^). *M. intracellulare* was the most common causative species in the MAC cohort (83%), and nodular bronchiectasis was common (60-90%). Collectively, these characteristics are typical in of an NTM-PD setting (12).

One patient in the active MAC cohort and one patient in the PostTx MAC cohort had a prior history of NTM infection (**Table 1**). A three-year follow-up of the active NTM-PD cohorts showed 42-65% of patients responded to therapy, 12-25% went on to persistent infection, and 10-14% subsequently relapsed (**Table 1**).

### Immune cell ratios are poor biosignatures for NTM pathology

To better understand NTM immunopathology, we examined the immune compartment during and after NTM-PD. This involved the optimization and implementation of three 12-16 colour flow panels covering αβ and γδ T cell subsets, mucosa-associated invariant T cell (MAIT) subsets, NKT cell subsets, NK cell subsets, B cell subsets and monocyte subsets. Representative gating strategies of each panel are shown in (**Supplemental Figures 3**–**6**). Despite comprehensive multiparametric coverage, we could find no significant univariate differences in immune cell subset percentages between NTM-PD cohorts and controls. This included MAIT cells, which decrease in frequency across many diseases.

### Exhaustion marker expression patterning correlates with NTM-PD staging

**Figure 2:**
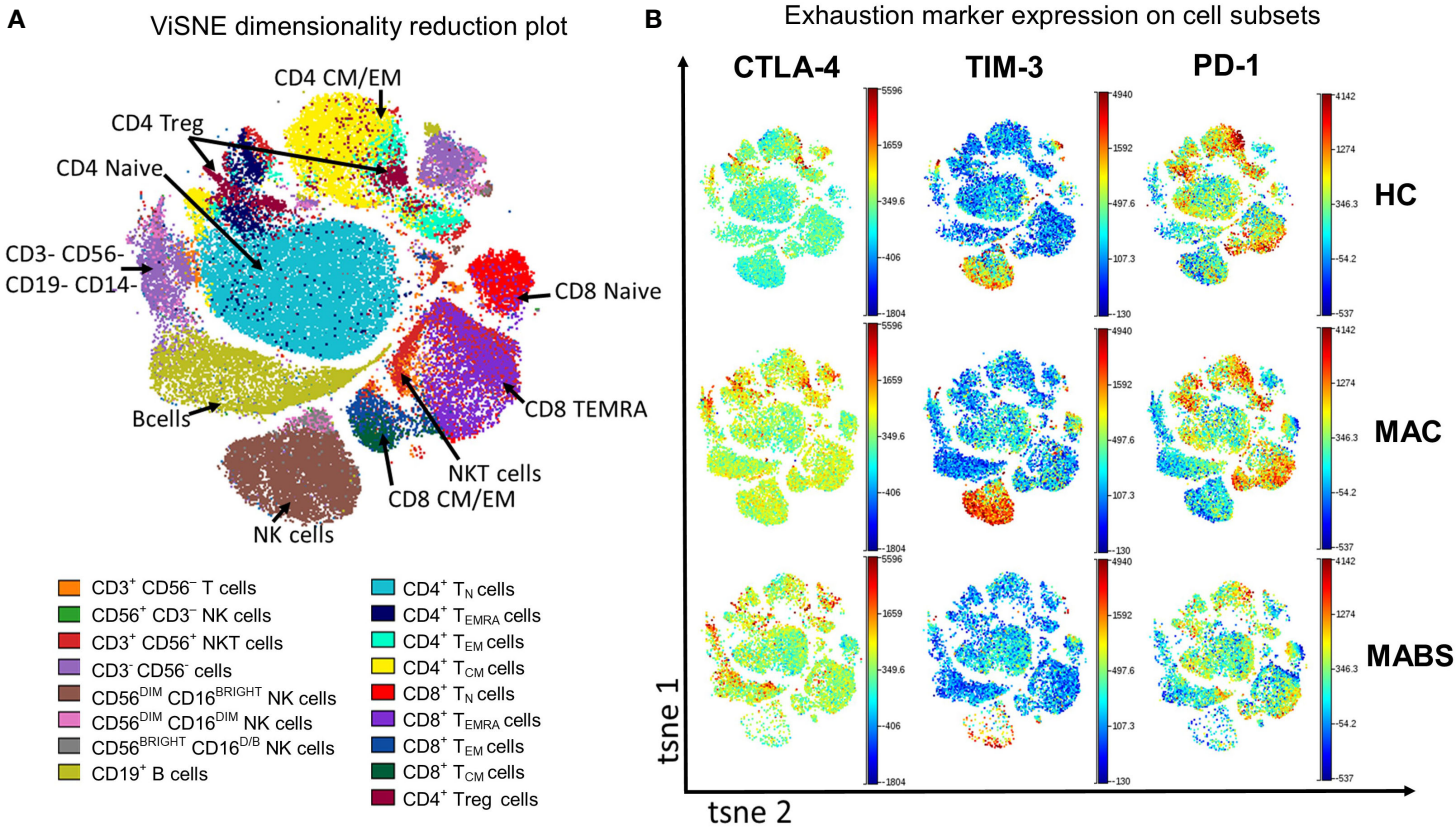
Peripheral blood phenotyping analysis shows increased TIM-3 and CTLA-4 in patients with active MAC and MABS infection. **(A)** viSNE was used to separate immune subsets based on phenotype. T_N_ – naïve T cells T_CM_ – central memory T cells, T_EM_ – effector memory T cells, T_EMRA_ – effector memory T cells with RA phenotype. **(B)** Representative ViSNE plots from HC, active MAC patient, and active MABS patient are shown. ViSNE plots are coloured by the level of expression of CTLA-4, TIM-3, and PD-1.

We next examined immune checkpoint dynamics across the cohorts. Using a tool that converts high-dimensional cytometry data into 2D space (viSNE), immune subsets were separated into their respective lineages (**Figure 2A**). The viSNE analysis showed elevated CLTA-4 expression on multiple effector subsets during active MABS and active MAC infection (**Figure 2B**). TIM-3 expression was increased during active MAC infections, particularly on NK cells, while PD-1 showed slight variation with controls (**Figure 2B**).

We next used a similar tool that provides simplified presentation and evaluation of high-dimensional cytometry data (Simplified Presentation of Incredibly Complex Evaluations –’SPICE’) to examine for heterogeneity in immune exhaustion receptor expression. A total of 28 exhaustion ‘fingerprints’ were determined across αβ T cells, NKT, NK cell and monocytes which patterned by cohort (**Table 2**). Consistent with ViSNE analysis, CTLA-4^+^ CD4^+^ T cells were elevated in active MABS patients, and elevated TIM-3 levels were observed in CD8^+^ T cell and NK cell subsets, particularly in MAC infection. PostTx MAC patients showed relatively few differences compared with other NTM-PD cohorts. Of note, exhaustion marker fingerprints in persistently infected patients were remarkably like HC, indicating that they were ‘healthy’ by immune profiling. Bronchiectasis controls differed from HC only with elevated CTLA-4^+^ on CD4^+^ T_EMRA_ cells and CD4^+^ Tregs. Of note, exhaustion fingerprints were not restricted to T cells but also present in NKT, NK cell and monocyte subsets.

**Table 2:** SPICE analysis-based exhaustion marker fingerprint on immune cell subsets in patient cohorts compared to healthy controls.

Cell type	Subset	CTLA-4	TIM-3	PD-1	Active MAC p value	PostTx MACp value	Active MABSp value	Persist infection p value	Bronch control p value
**CD3^+^ CD4^+^ T cells**	T_CM_	+	–	–	(+) 0.039		(+) 0.001		
	T_TEM_	–	–	+			(-) 0.029		
	T_EM_	–	+	–	(+) 0.009				
	T_EM_	–	–	+			(-) 0.033		
	T_EM_	+	–	–			(+) 0.002		
	T_EMRA_	+	+	–			(+) 0.018	(+) 0.014	(+) 0.002
	T_EMRA_	+	–	–			(+) 0.002		(+) 0.047
	T_regs_	+	–	–			(+) 0.018		(+) 0.031
**CD3^+^ CD8^+^ T cells**	T_CM_	+	–	–			(+) 0.019		
	T_TEM_	–	+	+		(+) 0.016		(-) 0.036	
	T_TEM_	–	+	–	(+) 0.024				
	T_EM_	–	+	–	(+) 0.002	(+) 0.016			
	T_EM_	–	–	+	(-) 0.033				
	T_EMRA_	–	+	–	(+) 0.005				
	T_EMRA_	–	–	+			(-) 0.013		
**CD3^+^ CD4^–^ CD8^–^ T cells**		–	+	–	(+) 0.001	(+) 0.002	(+) 0.018		
**CD3^–^ CD56^+^ NK cells**	CD56^bright^ CD16^dim^/^bright^	–	+	–	(+) 0.006		(+) 0.026		
	CD56^bright^ CD16^dim^/^bright^	–	–	–	(-) 0.007		(-) 0.035		
	CD56^dim^ CD16^bright^	–	+	–	(+) 0.021				
	CD56^dim^ CD16^bright^	–	–	–	(-) 0.020				
	CD56^dim^ CD16^dim^	+	+	–				(+) 0.029	
	CD56^dim^ CD16^dim^	–	+	–	(+) 0.035				
	CD56^dim^ CD16^dim^	–	–	–	(-) 0.023				
**CD3^+^ CD56^+^ NKT cells**		–	–	+			(-) 0.024		
**Monocytes**	CD14^+^ CD16^–^ classical	–	+	–	(-) 0.010				
	CD14^+^ CD16^–^ classical	–	–	–	(+) 0.012				
	CD14^dim^ CD16^+^ non- classical	–	+	–	(+) 0.024				
	CD14^dim^ CD16^+^ non-classical	–	–	–	(-) 0.020				

T_CM_: T central memory cells.

T_TEM_: Effector memory T cells.

T_EM_: T effector memory cells.

T_EMRA_: T effector memory cells with RA phenotype.

(+) Significant increase in cell percentage compared to healthy controls.

(-) Significant decrease in cell percentage compared to controls.

Red and blue shading indicates the direction and significance power. Red is increased relative to controls, and blue is decreased.

**Figure 3:**
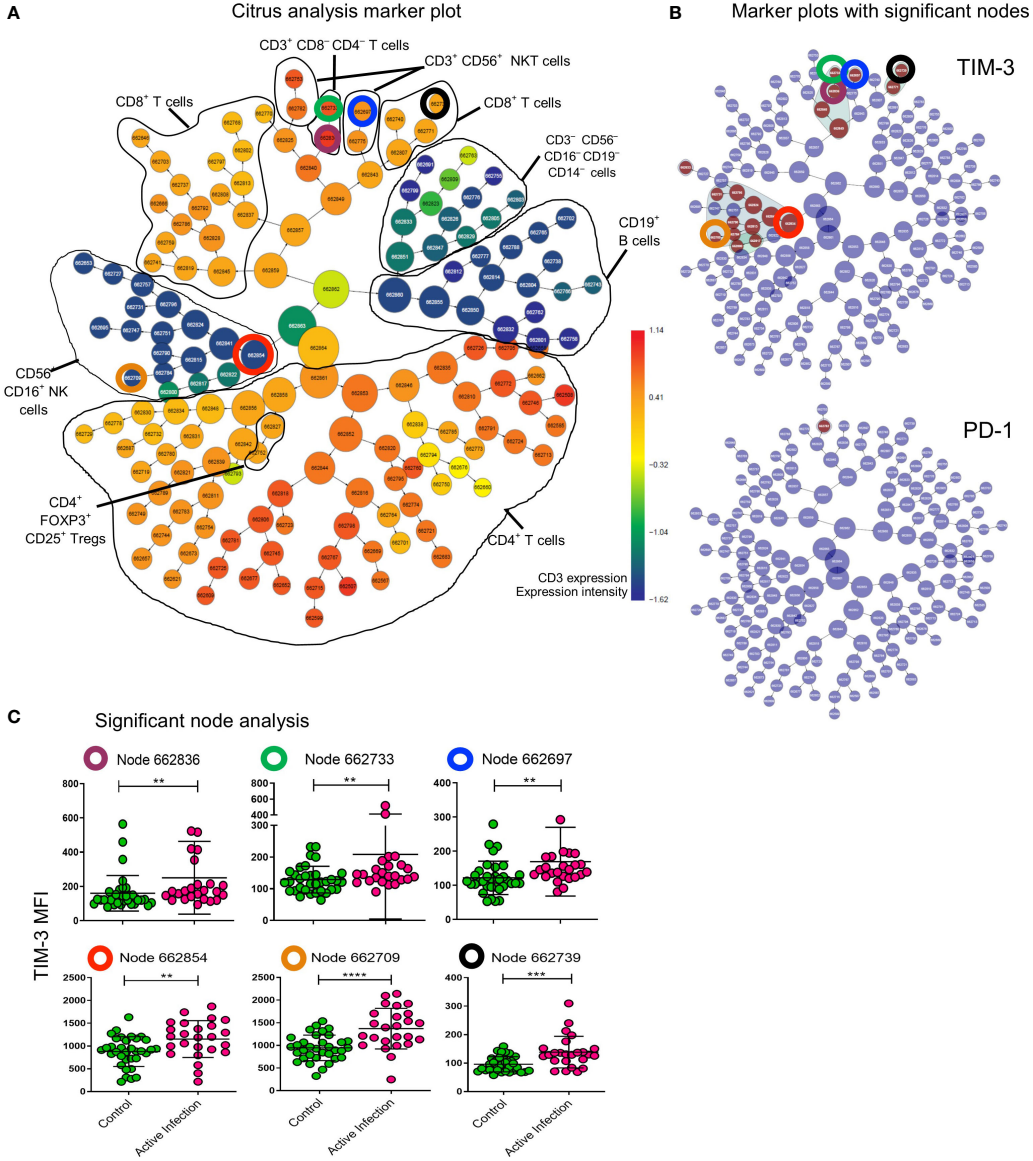
CITRUS analysis defines effrctor cell nodes that upregulate TIM-3 during active infection. All cells of lymphocyte gate in patients with Active infection (active MAC and active MABS) and controls (BronchC and HC) were analysed with CITRUS (Becman Coulter). **(A)** Marker plot showing all cells (central node) sequentially divided into daughter nodes based on dissimilarity of expression of lineage markers specified in the analysis setup (coloured by CD3 expression as heatmap). Black lines – cell subsets identified based on other marker plots not shown here. The total lymphocyte gate is represented in this plot. **(B)** The same marker plot as shown in **(A)** are coloured by cell nodes (brown) that have significantly different expression of TIM-3 and PD-1 to controls. CTLA-4 was not differentially expressed in this analysis. Nodes that were analysed further are highlighted with coloured circles. Corresponding circles in the marker plot show the lineage of the node **(A)**. **(C)** Nodes identified for further analysis are identified by the coloured circle and by node number. MFI of TIM-3 for the cells assigned to the given node for each sample is shown. Comparison between cohorts was performed by Mann-Whitney test. Maroon circle CD3^+^CD4^-^CD8^-^ T cell parent node (p=0.0039); Green circle subset of CD3^+^CD4^-^CD8^-^ T cells (p=0.0071); blue circle CD3^+^CD56^+^NKT cells (p=0.0014); red circle CD3^-^CD56^+^CD16^+^ NK cell parent node (p=0.0041); orange circle CD3^-^CD56^+^CD16^+^ NK cell daughter node (p<0.0001); black circle CD3^+^CD8^+^ T cells (p=0.0005). ****p < 0.0001, ***p < 0.001, **p < 0.01.

We next asked whether immune biosignatures could differentiate individuals with active mycobacterial infection (MAC or MABS) from individuals without a history of NTM pathology (HC and BronchC). Using the supervised machine learning tool (CITRUS), which probes for biomarkers using high-dimensional flow input data (**Figure 3A**), we identified cell clusters that had significant differences in TIM-3 and PD-1 expression in T cells and NK cell subsets associated with ‘active disease’ status (**Figure 3B**). Manual gating and statistical analysis of individual CITRUS discovery nodes (**Figure 3C**) verified CITRUS findings. Here, NTM-PD patients with active MAC infection showed elevated TIM-3 expression in all nodes (**Figure 3C**). These results verify the SPICE findings.

To probe for catastrophic failures in global immunity, PBMC were stimulated with a mitogen (αCD3/αCD28 antibodies) and tracked *in vitro* by flow cytometry and cytometric bead array (CBA). No differences in proliferation or effector function (IL-2, IL-4, IL-10, IL17, IFN-γ, TNF and CD107a) were found in CD4^+^ T cells, CD8^+^ T cells, NKT cells or NK cells T cells by SPICE analysis (**Supplementary Figure 1**).

### T cell gene signatures inform on NTM-PD staging

To validate the protein data (above) and probe for novel circulating biosignatures, particularly a signature for patients with persistent infection which had eluded us thus far, we next performed NanoString gene quantification on CD4^+^ and CD8^+^ T cells (33). For this analysis, HCs and BronchC were combined into a single control group as both multivariate Principal Component Analysis (PCA) and univariate analysis (*t*-test, adjusted for multiple comparisons FDR>0.05 all genes) showed no difference between cohorts

**Figure 4:**
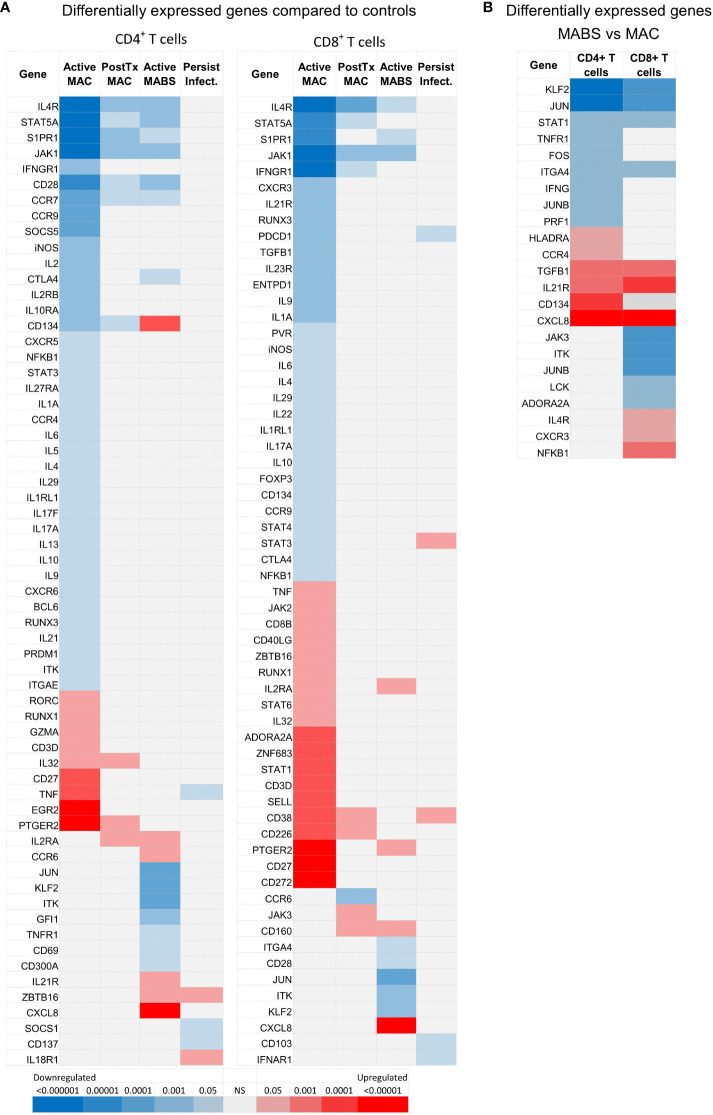
NanoString-based gene expression levels in CD4^+^ and CD8^+^ T cells in NTM-PD. **(A)** Statistical heatmap of upregulated (red) and downregulated (blue) genes in patients with active MAC, PostTx MAC, active MABS or persistent infection (MAC or MABS) compared to the control group (BronchC and HC). **(B)** Statistical heatmap of upregulated and downregulated genes in patients with active MABS infection compared to patients with active MAC infection. Independent *t*-tests were performed using GMine with correction for multiple comparisons (FDR). Significance (p-value) is shown by colour intensity.

T cell gene expression patterns differed by disease staging, and, akin to flow data, patients with active MAC or MABS infection showed the most divergent biosignatures (**Figure 4A**). In contrast, gene patterns in patients with persistent infection closely mirrored controls. Active MAC infection and active MABS infection showed the highest degree of deviation and specific T cell gene patterns were identified that associated with infecting species (**Figure 4B**). Interestingly, PD-1, CTLA-4 and TIM-3 were not among the differentially expressed genes, however, it is known that poor correlation exists between gene and protein abundance in T cells (34). Nonetheless, as digital gene profiling could better differentiate NTM-PD cohorts, we pursued this platform to search for circulating immune biomarkers of infection.

**Figure 5:**
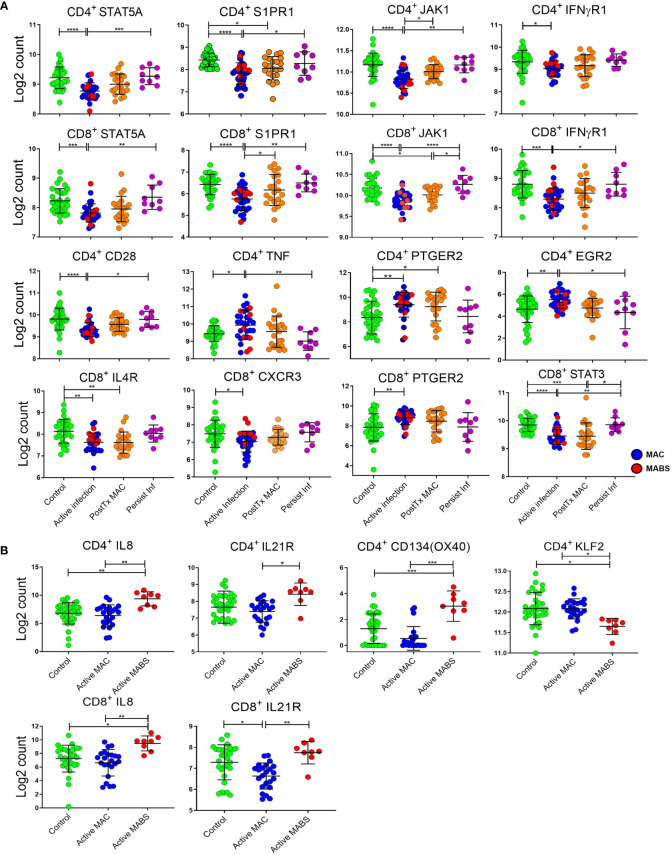
Relative expression of individual immune genes in CD4^+^ and CD8^+^ T cells. **(A)** Expression, as log2 counts, of immune genes that differentiate active infection [MAC (blue) and MABS (red)] from Controls [BronchC and HC (green)]. Levels of PostTx MAC (orange) and Persistent Infection (purple) and significance of comparisons are shown. **(B)** Expression of immune genes differentiating infecting species (MAC versu MABS) in active infection are shown as log2 counts of RNA copy numbers. Mean, and SD are shown in black bars. Comparisons between cohorts were performed by ANOVA with Tukeys *post hoc* test. P values were adjusted for multiple comparisons ****p < 0.0001, ***p < 0.001, **p < 0.01, *p < 0.05.

We first investigated circulating T cell gene biomarkers in NTM-PD patients before treatment. For this analysis, active MAC and active MABS patients were combined and defined as having ‘active infection’ and compared with the PostTx MAC, persistent infection and control cohorts. **Figure 5A** shows the log2 counts of the top eight most differentially expressed genes between patients with active infection (MAC and MABS) and controls in CD4^+^ and CD8^+^ T cells (FDR<0.05). Significant suppression of STAT5A (CD4^+^ p<0.0001; CD8^+^ p=0.0004) and JAK1 (CD4^+^ p<0.0001; CD8^+^ p<0.0001) as well as S1PR1 (CD4^+^ p<0.0001; CD8^+^p<0.0001) and IFNγR1 (CD4^+^ p=0.0363; CD8^+^ p=0.0001) were seen. In contrast, S1PR1 expression was significantly increased in persistent infection compared to active infection in both CD4^+^ (p=0.043) and CD8^+^ (p=0.0036) T cells, as were STAT5A levels (CD4^+^ p=0.0006; CD8 p=0. 0023) JAK1 levels (CD4^+^ p=0.0011; CD8^+^ p<0.0001) and CD8^+^ IFNγR1 levels (p=0.0217).

PTGER2 was significantly upregulated in both CD4^+^ (p=0.0039) and CD8^+^ (p=0.0086) T cells in active infection, as were CD4^+^ TNF expression (p=0.0364) and CD4^+^ EGR2 expression (p=0.0069). CD4^+^ TNF expression was significantly lower in persistent infection compared to active infection (p=0.0058) as was CD4^+^ EGR2 (p=0.020).

### T cell gene signatures inform on disease-causing species in NTM-PD

Next, differentially expressed genes across active MAC and active MABS patients were examined for potential species-specific immune signatures (**Figures 4B** and **5B**). Active MABS infection showed a significant increase in IL-8 (CD4^+^ p=0.001; CD8^+^ p=0.007) as well as IL-21R (CD4^+^ p=0.0152; CD8^+^ p=0.010) compared to MAC infection. However, these IL-21R levels were comparatively high in MABS infection due to suppression in MAC infection rather than an increase associated with MABS. IL-8 expression was also significantly higher in active MABS infection compared to control cohorts for CD4^+^ T cells (p=0.004) and CD8^+^ T cells (p=0.05), indicating a strong association with MABS species infection. Moreover, we identified a significant increase in CD4^+^ CD134 (OX40) in MABS infection (p<0.0001). KLF2 in CD4^+^ T cells was the only marker that was suppressed in MABS infection compared to both MAC patients (p=0.03) and controls (p=0.02).

### Forecasting clinical outcomes in NTM-PD using immune-based signatures

**Figure 6:**
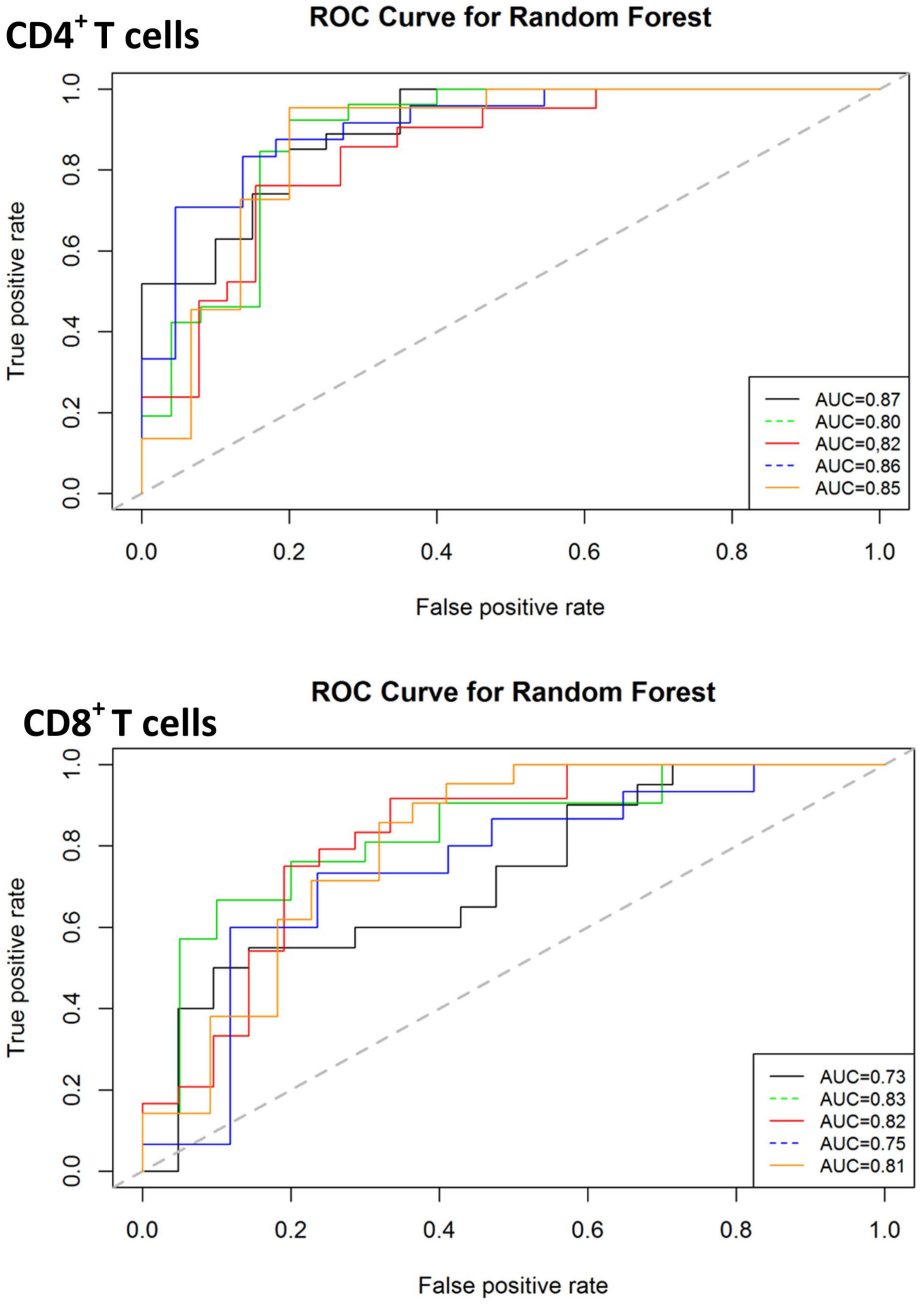
Classification performance of predictive models on validation data sets for diagnosis of active infection. The ROC curves for predictive model accuracy. Random forest models developed on 70% of test data sets were validated on 30% validation datasets. The process was repeated five times. AUC – Area under the curve.

Given that both proteomic and transcriptomic landscapes changed based on disease status, we next used supervised random forest modelling to test the accuracy of these immune-based signatures for identifying NTM-PD and for identifying the disease-causing species. Receiver operated characteristic (ROC) curves for each of the five iterations of modelling carried out (on randomly selected training and validation data sets) reported an accuracy ranging from 0.75 to 0.95, sensitivity from 0.67 to 1.0 and specificity from 0.78 to 1.0 for the CD4^+^ T cell data and accuracy ranging from 0.73 to 0.91, sensitivity from 0.70 to 1.0 and specificity from 0.66 to 1.0 for the CD8^+^ T cell data (**Figure 6**). Assuming genes with higher variable importance play a greater role in prediction, a nine gene signature consisting of the most frequently top-ranked genes for CD4^+^ T cells (> 3 times/5 iteration) was identified, comprising S1PR1, IL4R, STAT5A, JAK1, CD28, SOCS5, IL10RA, GFI1 and BCL6. All these genes were significantly downregulated in active infection. The predictive performance of the nine gene signature to correctly classify actively infected individuals based on CD4^+^ T cell gene expression data was evaluated by LOOCV and returned an accuracy of 88%, sensitivity of 81%, and specificity of 93%. For CD8^+^ T cells, a seven-gene signature was identified, comprising JAK1, S1PR1, IFNγR1, STAT5A, PTGER2, CD27 and CXCR3. Only two genes (PTGER2 and CD27) were significantly upregulated in active infection, while the remaining five were downregulated. This seven-antigen signature achieved an accuracy of 84%, sensitivity of 81% and specificity of 86%, correctly classifying individuals as having active NTM infection.

The most influential genes in differentiating infecting species were JUN, IL21R, KLF2 and CD134 in CD4^+^ T cells and IL21R, TGFB1, HLA-DR and IL1A in CD8^+^ T cells (**Supplemental Figure 2**). A second supervised random forest forecasting analysis to determine whether we could indirectly identify the causative species based on gene expression data revealed 95% accuracy using CD4+ T cell data and 93% accuracy using CD8+ T cell data. This forecasting, developed on 70% of the data and validated on 30%, also requires further authentication at independent sites but provides a basis for using the immune system as an indirect diagnostic platform for NTM-PD disease.

### The functionality of antigen-specific immunity correlates with NTM-PD staging

**Figure 7:**
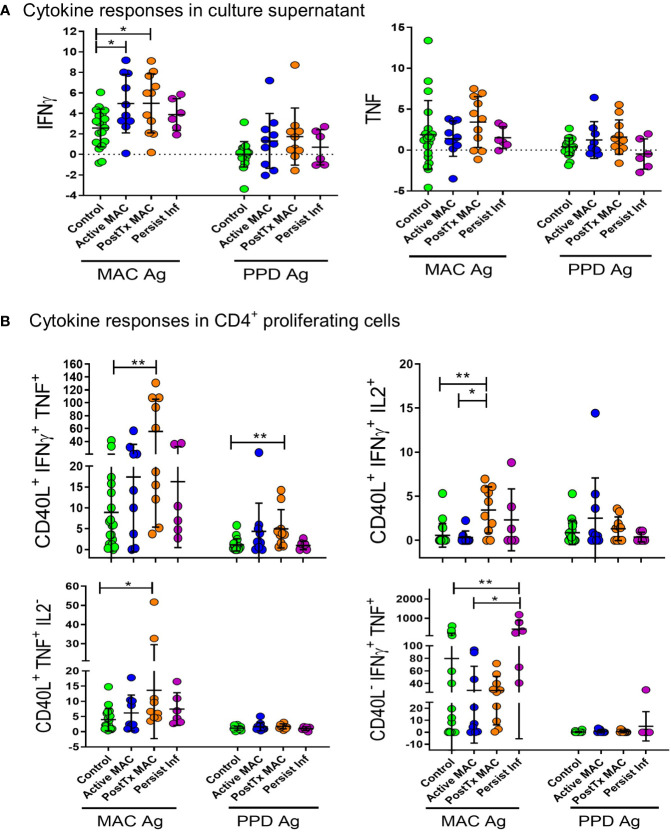
Antigen stimulation of peripheral blood mononuclear cells. **(A)** Supernatant IFN-γ and TNF cytokine levels after antigen stimulation are presented as log2 of fold change over unstimulated control. Antigens used for stimulation were heat-killed organism crude extract (MAC antigen) and clinical-grade PPD. Comparisons were performed by ANOVA with Tukeys *post hoc* comparisons. **(B)** Antigen-specific cytokine responses were quantified by proliferating CD40L^+^CD4^+^ T cells stimulated by heat-killed MAC Ag or PPD. Percentage of positive cells are shown as fold change relative to unstimulated controls. A Kruskall Walliis test with Dunns *post hoc* was performed for comparitive analysis. **p < 0.01, *p < 0.05.

Given the global immune dysfunctions described above, we next asked whether antigen-specific immunity was affected in NTM-PD patients. Here, we quantified antigen-specific cytokine responses from patient PBMC against crude MAC antigen (heat-killed) or clinical-grade purified protein derivative (PPD). Clinical-grade PPD was included for cross-validation and robustness, given crude antigen likely contain mitogens and endotoxins. We found elevated IFN-γ responses in active MAC patients and PostTx MAC patients stimulated with crude antigen (**Figure 7A**), indicating that the IFN-γ axis appears to be functionally intact in these individuals. This trend was also present in PPD stimulated samples (**Figure 7A**). Analysis of CD40L^+^ antigen-specific proliferating cells in PostTx MAC patients showed CD40L^+^ IFN-γ^+^ TNF^+^ CD4^+^ T cells were significantly higher compared to healthy controls for crude antigen (p=0.006) and PPD (p=0.007) (**Figure 7B**). Moreover, proliferating CD40L^-^ IFN-γ^+^ TNF^+^ CD4^+^ T cells were significantly higher in patients with persistent infection (**Figure 7B**). This subset was increased when compared to both control subjects (p=0.006) and patients with active infection (p=0.05). This was significant evidence of an immune biosignature for persistent infection.

### Immune checkpoint modulation enhances T cell efficacy

The regulatory approved immune checkpoint inhibitor nivolumab, which blocks the PD-1 signaling axis, was next examined in combination with antigen stimulation assay to determine whether NTM immunity could be augmented by reversing this exhaustion pathway.

**Figure 8:**
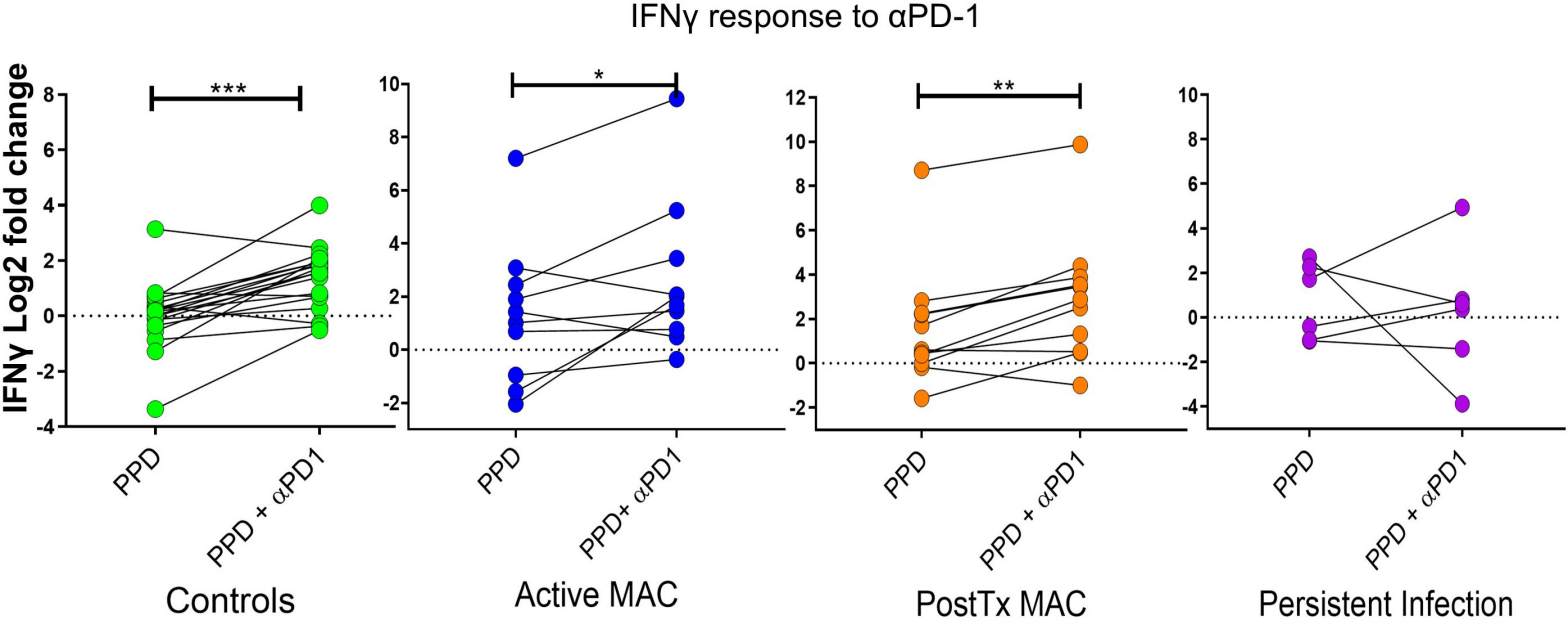
IFN-γ response to treatment with nivolumab (αPD-1). Dot plots show IFN-γ response (measured by cytometric bead array) to PPD in patients and controls with and without αPD-1 treatment. IFN-γ levels are shown as log2 fold change over level of unstimulated wells. A Wilcoxon test was performed. ***p < 0.001, **p < 0.01, *p < 0.05.

We assessed IFN-γ levels relative to unstimulated controls using PPD antigen stimulation and PD-1 blockade (**Figure 8**). PD-1 blockade significant augmented IFN-γ production in the active MAC cohort (p=0.043, R^2 =^ 0.38). PostTx MAC cohort and control subjects showed stronger increases in IFN-γ (p=0.0025, R^2 =^ 0.612 and p<0.0001, R^2 =^ 0.589). In contrast, patients with persistent infection showed no significant change in IFN-γ levels in response to αPD-1 treatment. In all experiments performed thus far, this, together with a faint signal in checkpoint fingerprinting and the finding of elevated CD4^+^CD40L^-^ IFNγ^+^ TNF^+^ production, were the only test parameters that showed a difference between persistent infection and controls. The isotype control had no significant effects across all cohorts (not shown), indicating αPD-1 blockade was successful.

These data collectively provide insight into the functional immune landscape and immune failures in NTM-PD. We found that a protective response was associated with proliferating, antigen-specific IFN-γ^+^ TNF^+^ CD4^+^ T cell cells, which were prominent in patients who cleared the infection. In persistent infection, an increase of proliferating IFN-γ^+^ TNF^+^ CD4^+^ T cells was observed; however, these cells were CD40L^-^ negative, suggesting immune dysfunction initiated by the pathogen or underlying failure in basic immune function. Akin to the above profiling, there was no αPD-1 augmentation in persistently infected patients relative to controls, while active MAC and PostTx MAC patients augmented IFN-γ^+^ secretion during PD-1 blockade. Interestingly, we identified noticeable populations of mycobacterial-specific effector cells in healthy individuals indicating past and likely repeated challenges during the human life course. Healthy individuals could also augment the NTM response with αPD-1 blockade.

## Discussion

The increasing prevalence of NTM-PD poses a significant burden to global health (9, 17). Thus far, the immune mechanisms underlying NTM-PD are largely unknown. We investigated the immune compartments of a large and well characterized NTM-PD cohort using high-dimensional immune phenotyping to address this gap in knowledge.

Multiparametric flow analysis revealed immune checkpoint marker ‘fingerprints’ that grouped by NTM-PD stage and causative species. Specifically, MAC infection triggered the upregulation of TIM-3 on CD8^+^ T cells and NK cells, whereas MABS infection triggered the upregulation of CTLA-4 on multiple immune subsets. The second observation validates our preceding work suggesting immune dysfunction and T cell exhaustion in individuals with MABS NTM-PD (35). This study revealed several new and intriguing findings. Firstly, we observed the immune system customizing itself based on specific ‘threats’, even if they are evolutionarily related. Secondly, lung infection triggered global changes in the circulating immune compartment. These findings suggest that many activated immune cells are ‘spilling out’ into the circulation from the infected lung and/or the infected lung is secreting ‘danger signals’ alerting the entire immune network. Thirdly, in at risk individuals, numerous immune failures were observed in crucial effector lineages (Tregs, T cells, NKT cells, NK cells and monocytes).

Importantly, in addition to the upregulation of exhaustion markers, we defined a nine-gene signature in CD4^+^ T cells (S1PR1, IL4R, STAT5A, JAK1, CD28, SOCS5, IL10RA, GFI1 and BCL6) and a seven-gene signature in CD8^+^ T cells (JAK1, S1PR1, IFNγR1, STAT5A, PTGER2, CD27 and CXCR3) which could differentiate active NTM-PD infection from healthy individuals and bronchiectasis controls. This is advantageous in a clinical setting as many of the patients screened for active NTM infection have underlying airway inflammatory diseases such as bronchiectasis and COPD. The ability to identify active NTM-PD and the disease-causing species from a simple blood draw has potential. Firstly, given NTM are ubiquitous in the environment, diagnostic false-positive sputum cultures can occur through contaminated samples and commensal NTM temporarily residing in the upper airway (36). Thus, multiple positive respiratory cultures are required to satisfy microbiological criteria (37). Secondly, a venous blood draw is a rapid, non-invasive, low-risk procedure and included in the standard of care. Thirdly, NanoString profiling can be completed in hours compared with weeks for the gold standard of NTM microbiological culture and biochemical testing. In NTM-PD, the rapid identification of the disease-causing agent is highly advantageous, given that treatment regimens differ extensively between species (38). The ability of blood-based immunoprofiling to inform on causative species and guide treatment requires further investigation but could potentially guide treatment in smear-positive patients where pathology is present, but the disease-causing species may take weeks to obtain. Sanger sequencing-based and proteomic-based assays are in development for NTM detection; however, they suffer from the same limitation as NTM culture, namely false-positive results. Given blood-based immunoprofiling is an entirely different sensing/diagnostic perspective, this platform circumvents the problem of sample contamination.

Patients with active mycobacterial infection showed differential expression of many genes in CD4^+^ and CD8^+^ T cells. In both lineages, upregulated genes included PTGER2, CD27, CD272 and CD226, and downregulated genes included IFNγR1, JAK1, STAT5A and S1PR1. The biosignatures in PostTx MAC and Persist Inf patients indicate that the expression of these genes reverts to healthy controls profiles. However, some signatures remain even when patients are in disease remission. Counterintuitively, persistently infected NTM-PD patients showed curiously similar immune profiles to controls and lacked significant biosignatures in the circulating blood, suggesting the immune system in these individuals is ‘ignoring’ the danger in the lung. This cohort responded only weakly to antigen but responded to potent mitogen, suggesting intact effector functions but possible failures in pathogen sensing, immune system cross-talk or immune activation threshold.

The downregulation of IFNγR1 in CD4^+^ and CD8^+^ T cells in NTM-PD patients supports the genetic findings of IFNγR1 deficiency in Mendelian Susceptibility Mycobacterial Disease (MSMD) patients, who are at high risk of NTM-PD (39). Reversal of the suppression, seen in post-treatment patients, could restore CD8^+^ T cell IFN-γ mediated function. In addition, we have shown that antigen-specific IFN-γ responses appear to be intact in patients with active infection, though whether downstream signaling remains intact is unknown. This may explain why there is no consistent evidence of IFNγ deficiency in elderly patients with MAC infection and why trials of inhaled IFNγ did not show consistent results (9).

We found robust upregulation of TIM-3 on CD8^+^ T cells during MAC NTM-PD, suggesting this immune lineage plays a more prominent role in mycobacterial surveillance than previously appreciated. A previous whole blood gene expression analysis of NTM-PD patients showed differential expression of over 200 genes, including many immune-associated genes, including IFN-γ (40). While several other T cell genes were identified as differentially expressed, none of the patterns overlapped with the biosignatures identified in the present study, particularly S1PR1, STAT5A and JAK1.

IL-8 (CXCL8) is produced by macrophages and respiratory epithelial cells and, in TB, results in neutrophil recruitment, T cell recruitment and macrophage-mediated killing (41). In MABS infection, MABS variants induce an innate immune response through TLR2-mediated IL-8 secretion (42). The significant increase in IL-8 gene expression in both CD4^+^ and CD8^+^ T cells together with increased ex vivo IL-8 protein expression (data not shown) show that T cells are also a significant source of IL-8 during MABS infection. Elevated and continuous IL-8 secretion would explain the strong neutrophilic influx in MABS NTM-PD (43, 44). Whether IL-8 levels could be used for diagnostics or corrected using immunotherapy requires further investigation.

While robust immune biosignatures were found in active NTM-PD cohorts, only weak signals were identified in post-treatment disease remission and persistent infection cohorts. Patients in remission showed biosignatures that were ‘in-between’ active pathology and controls, showing that the immune system returns to partial but not complete homeostasis post-infection, suggesting underlying immune failures. It is possible that once an infection is established, true sterilising immunity does not occur often or quickly. This would explain why some patients are at high risk of recurrent infections.

Antigen-specific CD4^+^ T cells that produced both IFN-γ and TNF were hallmarks of patients in disease remission. This biosignature was consistent for crude antigen stimulation and PPD stimulation and is the first report of antigen-specific T cell polyfunctionality in MAC NTM-PD. Here, antigen-specific CD4^+^ T cells, which were IFN-γ^+^, TNF^+^ and/or IL2^+^ were elevated in PostTx MAC patients compared to controls and patients with active disease. These findings verify the prevailing model, where the Th1 response involving IFN-γ, IL-2 and TNF is required for mycobacterial detection and eradication. The difficulty in evaluating antigen specific T cell responses in the absence of specific tetramers is highlighted. Many factors, including cellular stress during culture conditions and impurities in antigens used can affect cell responses (45). We believe these extraneous factors account for the cellular responses seen even in the control samples, though some individuals may have previous NTM exposure as well, given how ubiquitous these organisms are in the environment. To identify the response to the specific antigen, over and above the culture conditions, without endotoxin effects, we primarily focused on the responses to PPD as fold change above the untreated control well for each sample.

Given the exhaustion marker, PD-1 was detected on the surface of multiple effector immune subsets in NTM-PD patients, and αPD-1 therapy has shown great potential in reversing immune exhaustion, we next blocked the PD-1 axis with nivolumab to ask if effector cells in NTM-PD patients could be functionally rescued. αPD-1 treatment augmented antigen-specific IFN-γ secretion in all patient and control cohorts except in persistently infected patients. Elevated IFN-γ secretion is consistent with the findings of Shu et. al., which showed a rise in IFN-γ levels in MAC antigen-stimulated NTM-PD samples when the PD-1 axis was blocked (24). Together, these data suggest effector cells from active MAC and PostTx MAC patients can be rescued from exhaustion. Of note, αPD-1-driven T cell augmentation was inconsistent in persistently infected patients, indicating alternate checkpoint/s may be involved (46), possible αPD-1 resistance or T cell senescence (47).

PD-1, CTLA-4, and TIM-3 blockade reduced disease severity in a mouse parasite infection model but could not provide sterilizing immunity or reverse susceptibility to second lethal infection (48). Trials of checkpoint blockade against six human pathogens are currently underway (23). The finding that persistently infected patients have proliferating, IFN-γ^+^ TNF^+^ CD40L^-^ CD4^+^ T cells in response to antigen and mitogen suggests that effector cells are not permanently offline and capable of rescue. Collectively, these data show TIM-3 and CTLA-4 play more dominant roles than PD-1 in mycobacterial infection and that targeting these checkpoints may be warranted. Indeed, TIM-3 blockade has shown efficacy in TB control in mice (22). Whether this can be mirrored in humans is unknown. It is important to highlight the time-sensitive nature of checkpoint inhibitor therapy. Specifically, treatment too early may be detrimental, and treatment too late, unresponsive (49).

PD-1, CTLA-4 and TIM-3 are upregulated on T cells during HIV, HBV, HCV, herpesviruses and cancer, resulting in altered metabolism, epigenetics, and responsiveness to cytokines (46). Our previous research shows CTLA-4 is upregulated on T cells during NTM-PD (35). Counterintuitively, while PD-1 expression is elevated on T cells during mycobacterial infection (7), receptor blockade can have adverse events in mycobacterial infection, including increasing TB sensitivity in mice (50), exacerbating TB in primates (51) and reactivation *in vitro* and cancer patients (7). Additionally, patients on PD-1 blockade therapy have an increased risk of TB pathology (52). However, given the major differences in immune configurations and ‘immune chatter’ during infection with even closely related pathogens, the efficacy of PD-1/PD-L1 inhibitors in NTM-PD is unknown and requires additional preclinical and clinical investigation.

T cells from TB patients show elevated PD-1 and TIM-3 that positively correlates with disease severity (53). One study showed elevated PD-1 and PD-L1 on circulating T cells during MAC infection (24). Our CITRUS analysis revealed PD-1 was elevated on specific T cell subsets during MAC infection but not to the same degree as the previous study. This study compared patients to only young, healthy individuals with normal pulmonary function, whereas our study included matched controls with chronic pulmonary pathology.

A surprise finding was the major role of TIM-3 in NTM pathology. TIM-3 is a less well-known member of the immune checkpoint family expressed on T cell, Treg cell, NKT cell, NK cell, dendritic cell, myeloid cell, and mast cell subsets (results and (54)) which can be membrane-tethered or secreted (9). Unlike PD-1 and CTLA-4, the cytoplasmic tail of TIM-3 lacks an inhibitory motif, and controversy exists over whether the receptor is co-inhibitory or co-stimulatory (9). In effector T cells, TIM-3 can be a negative regulator (55) or positive regulator (9), depending on the setting. For instance, soluble TIM-3 can suppress IL-2 secretion by T cells (9). In contrast, TIM-3 blockade can augment T cell proliferation and cytokine proliferation in HBV patient samples, but not in patients with weak T cell immunity (56). This observation draws similarities to the cohort of patients who failed to clear NTM from the lung. Overall, the function of TIM-3 is thought to prevent overreaction of the immune response.

There is promise in the redeployment of immune checkpoint therapies given co-PD-1/TIM-3 blockade synergise for superior T cell function (57). The first-in-class αTIM-3 antibody (sabatolimab) has received orphan drug status in humans, and clinical trials are underway for numerous cancers and patients who fail PD-1 blockade (58). Thus far, sabatolimab has exhibited an acceptable safety profile with positive pharmacokinetics/pharmacodynamics and is showing early promise in some cancers (59–61). Whether standalone TIM-3 blockade treatment or combination treatments are effective against NTM-PD(or infectious diseases in general) requires further preclinical and clinical trials. Overall, checkpoint receptor patterning is highly complex, and optimal checkpoint blockade efficacy may require targeting multiple immune cell subsets and multiple checkpoint processes at specific disease timepoints.

The general application of immune profiling for NTM-PD diagnostics/prognostics will require larger, multi-site cohorts before clinical deployment. Other unmet needs include examining pathogen-specific ‘immune chatter’ in other disease scenarios and *in vitro* testing of PD-1, CLTA-4, and TIM-3 blockade alone and combination.

Collectively, our results show that NTM-PD is associated with immune dysfunction and exhaustion in CD4^+^ and CD8^+^ T cells, NK T cells, NK cells and monocytes. This biosignature reverted towards normal with successful treatment. Persistently infected patients lacked peripheral immune signature, and exhaustion could not be reversed using PD-1 blockade. However, mitogen stimulation revealed no apparent failures in essential effector functions. Importantly, we show NTM-PD stage-specific and species-specific immune signatures. These signatures have a high potential in the development of rapid diagnostics and Point of Care (PoC) screening that can assist therapeutic decision making in the early stages of infection.

The immune system as a sensor for disease management has considerable potential. We have described MABS-specific immune signatures previously (35), and immune-based diagnostics are in development for other conditions (62–64). Whether immune sensing can improve NTM-PD diagnostics, prognostics and inform on new therapies requires further investigation.

## Data availability statement

The raw data supporting the conclusions of this article will be made available by the authors, without undue reservation.

## Ethics statement

The studies involving human participants were reviewed and approved by QIMR Berghofer (QIMRB) Human Research Ethics Committee (#P1479) Greenslopes Private Hospital HREC (12/12 & 14/14) James Cook University HREC (#H7010). The patients/participants provided their written informed consent to participate in this study.

## Author contributions

CR, SH, and MH participated in sample collection. CR performed the laboratory work with the assistance of KT, SH, VL, and PP in panel design. CR, MF and CP performed data analysis. CR, RT, SB and JM were involved in experimental design and planning. CR, KT and CP were involved in manuscript drafting, and CR, KT, DD, AK, SB, RT and JM, were involved in finalizing and editing the manuscript. All authors contributed to the article and approved the submitted version.

## Funding

Funding was provided by grants from the QIMRB Medical Research Institute and AITHM, James Cook University and the Rebecca L. Cooper Foundation for Medical Research (10509). The funders had no role in study design, data collection, analysis, or preparation of the manuscript. CR was supported by a University of Queensland International Postgraduate Scholarship and a James Cook University Post Graduate Research Scholarship. JM was supported by the Australian National Health and Medical Research Council (NHMRC) Career Development Level 1 and 2 Fellowships (1031652 and 1131732). DD was supported by NHMRC Principal Research Fellowship (1137285). MF was supported by NHMRC CJ Martin Fellowship (5121190). AK was supported by NHMRC Career Development Fellowship (1140709).

## Acknowledgments

We thank Rajiv Khanna for providing clinical-grade nivolumab. We thank the Queensland Mycobacterial Reference Laboratory for providing heat-killed clinical NTM isolates and the Gallipoli Medical Research Facility for the use of laboratory facilities. We thank the flow cytometry facility at QIMRB. Finally, we gratefully acknowledge all patients and healthy volunteers who participated in the study.

## Conflict of interest

The authors declare that the research was conducted in the absence of any commercial or financial relationships that could be construed as a potential conflict of interest.

## Publisher’s note

All claims expressed in this article are solely those of the authors and do not necessarily represent those of their affiliated organizations, or those of the publisher, the editors and the reviewers. Any product that may be evaluated in this article, or claim that may be made by its manufacturer, is not guaranteed or endorsed by the publisher.

## Supplementary material

Supplementary Figure 1 Polyfunctionality signatures of CD4^+^ and CD8^+^ T cells, NKT cells and NK cells in response to mitogen stimulation. Cytokines were quantified by stimulating PBMC with PMA and collecting the supernatant. Pie charts were generated by Boolean gating of cytokine positive populations and SPICE analysis. Pie slices represent the percentage of cells with a specific number of cytokine functions shown in the colour legend. Pie arcs represent the specific cytokines produced from effector cells, illustrated by colour coded legends separately for CD4^+^ T cells, CD8^+^ T cells, NKT and NK cells.

Supplementary Figure 2 Top differentiating genes for forecasting infecting species. Genes identified by random forest modelling as top predictor genes for differentiating active MAC versus active MABS infection in CD4^+^ and CD8^+^ T cells.

Supplementary Figure 3 Gating strategy for Immunophenotyping panel 1. A 16-colour immunophenotyping panel was designed and optimised to differentiate major lymphoid and monocyte cell subsets and quantify expression of the immune checkpoints CTLA-4, PD-1, and TIM-3. Gating strategy, cell subsets and functional markers are shown.

Supplementary Figure 4 Gating strategy for Immunophenotyping panel 2. A 12-colour immunophenotyping panel was designed and optimised to evaluate mucosal invariant T cells (MAITs) and γδ T cells with activation marker expression on each subset. Gating strategy, cell subsets and markers analysed are shown.

Supplementary Figure 5 Gating strategy for immunophenotyping and functionality analysis using panel 3. A 15-colour functionality panel was designed and optimised to evaluate six cytokines and a degranulation marker on multiple immune cell subsets. Gating strategy, cell subsets and functional markers are shown.

Supplementary Figure 6 Gating strategy for Antigen-specific proliferation and functionality testing panel 4. A 12-colour phenotyping and functionality testing panel that included proliferation and cytokine responses was designed and optimised. Gating strategy, cell subsets and functional markers are shown.
