# Supplementary figures and images for: Characterizing and correcting immune dysfunction in non-tuberculous mycobacterial disease
Source: Front Immunol. 2022 Nov 10;13:1047781. doi: 10.3389/fimmu.2022.1047781 (PMC9686449; doi:10.3389/fimmu.2022.1047781)

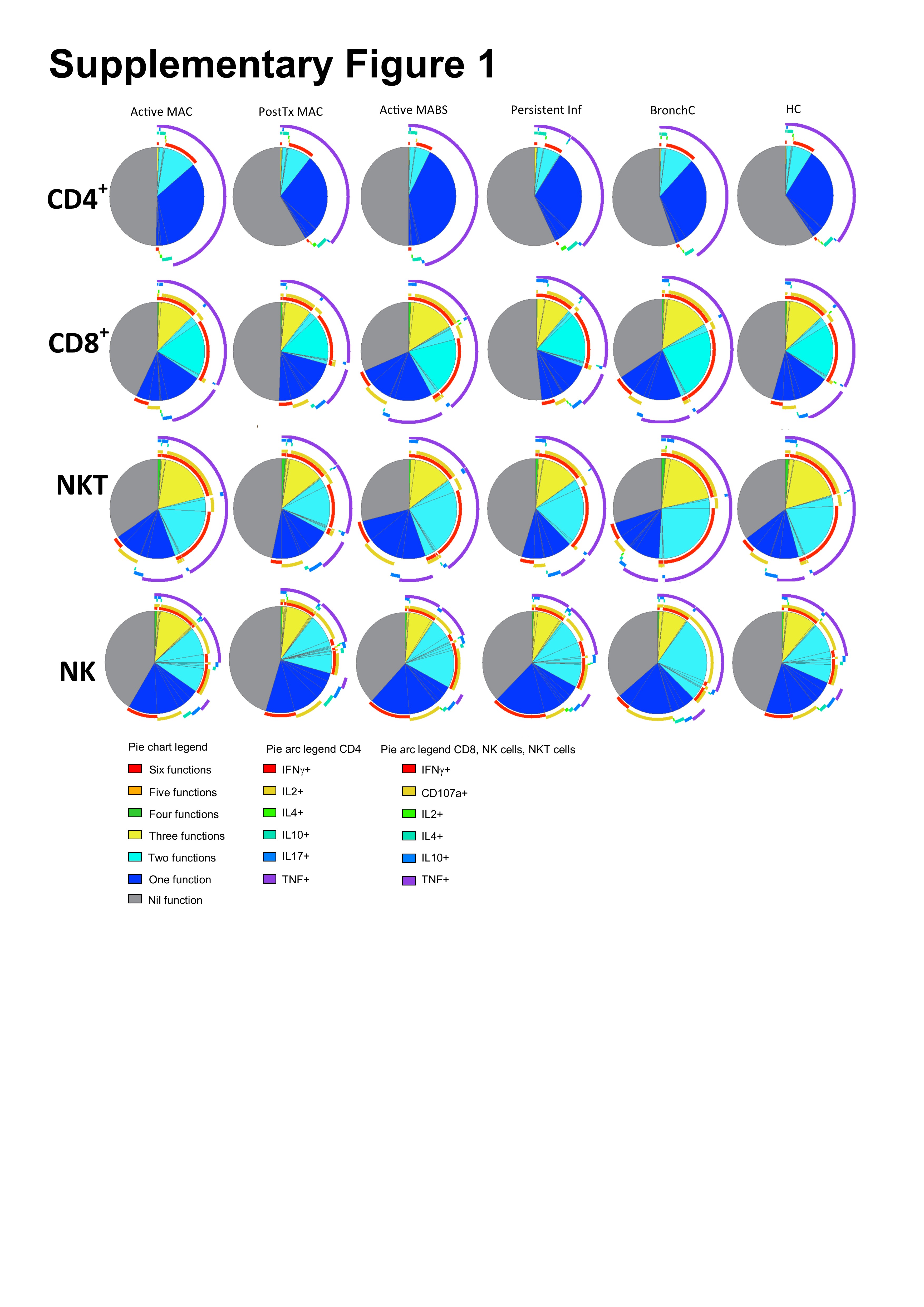

Supplement: Supplementary Figure 1 — Polyfunctionality signatures of CD4+ and CD8+ T cells, NKT cells and NK cells in response to mitogen stimulation. Cytokines were quantified by stimulating PBMC with PMA and collecting the supernatant. Pie charts were generated by Boolean gating of cytokine positive populations and SPICE analysis. Pie slices represent the percentage of cells with a specific number of cytokine functions shown in the colour legend. Pie arcs represent the specific cytokines produced from effector cells, illustrated by colour coded legends separately for CD4+ T cells, CD8+ T cells, NKT and NK cells. [file Image_1.jpeg]

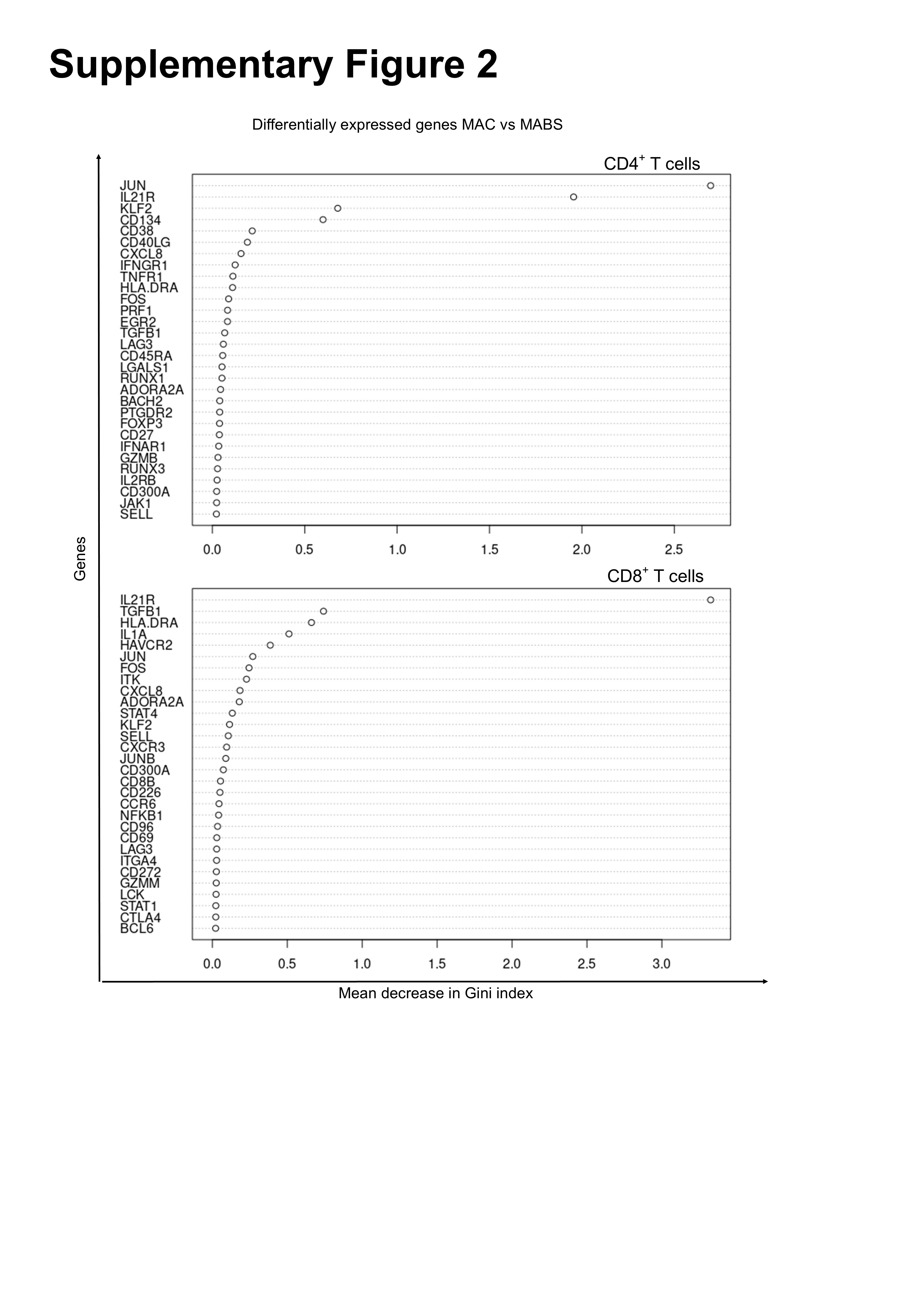

Supplement: Supplementary Figure 2 — Top differentiating genes for forecasting infecting species. Genes identified by random forest modelling as top predictor genes for differentiating active MAC versus active MABS infection in CD4+ and CD8+ T cells. [file Image_2.jpeg]

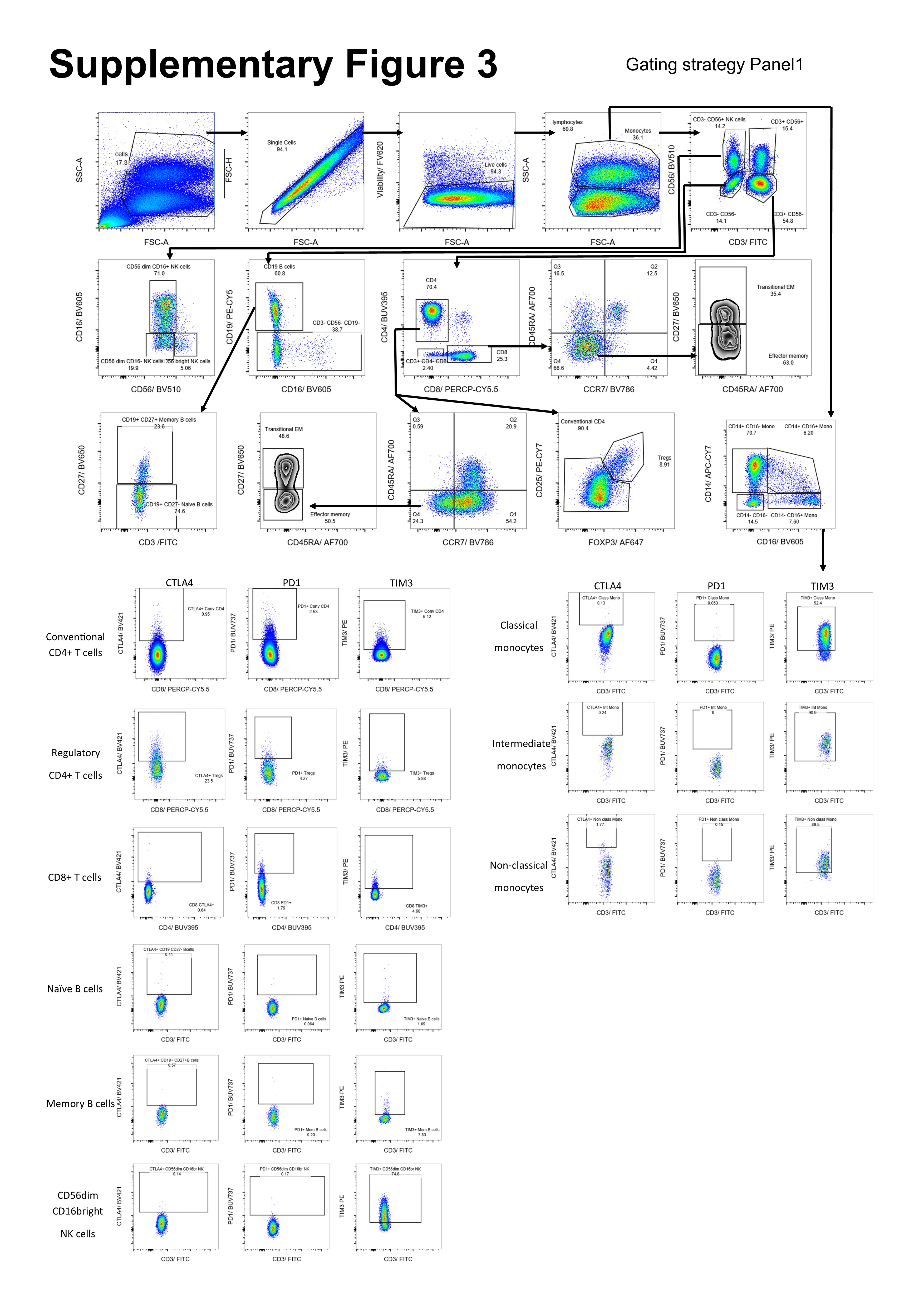

Supplement: Supplementary Figure 3 — Gating strategy for Immunophenotyping panel 1. A 16-colour immunophenotyping panel was designed and optimised to differentiate major lymphoid and monocyte cell subsets and quantify expression of the immune checkpoints CTLA-4, PD-1, and TIM-3. Gating strategy, cell subsets and functional markers are shown. [file Image_3.jpeg]

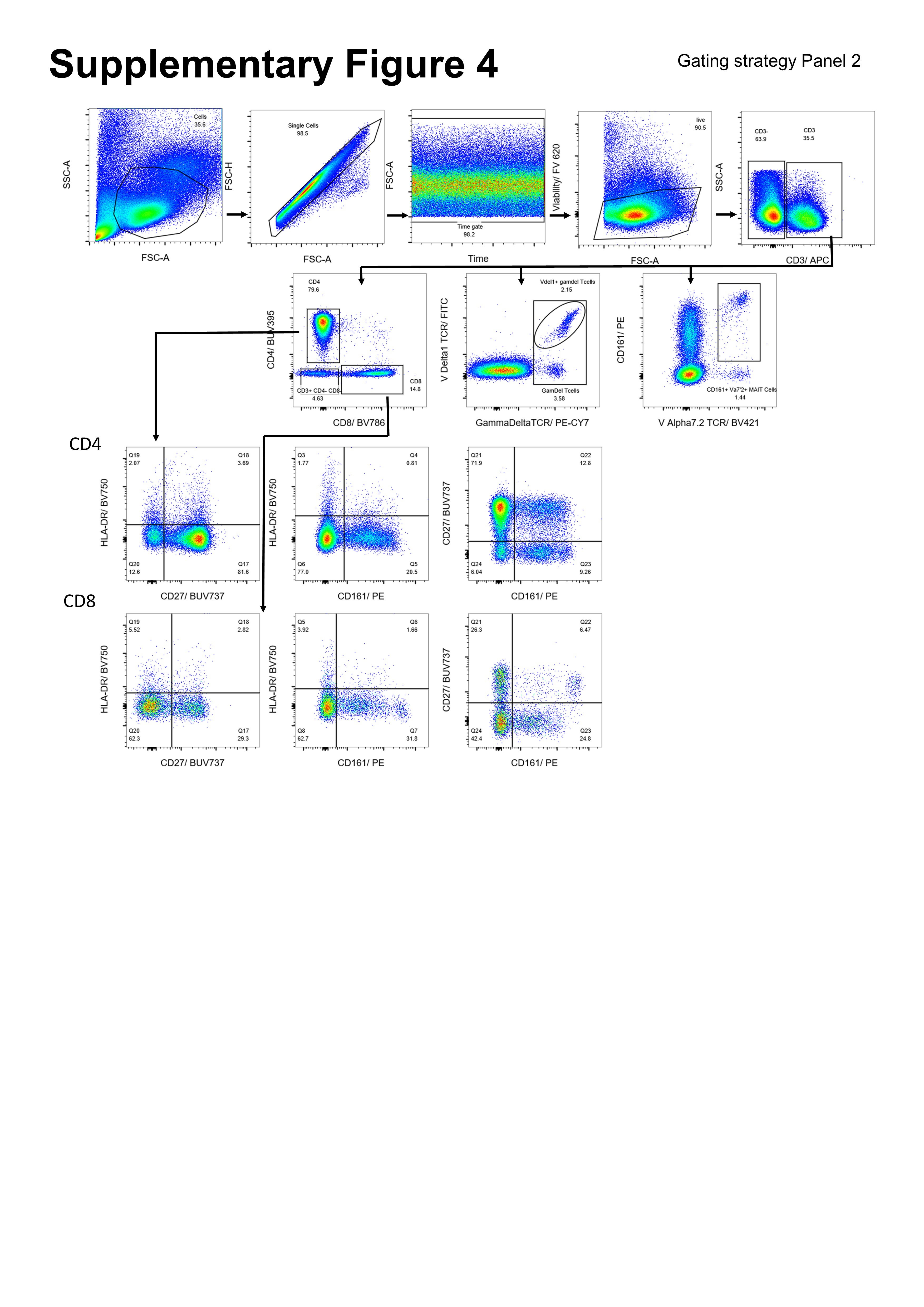

Supplement: Supplementary Figure 4 — Gating strategy for Immunophenotyping panel 2. A 12-colour immunophenotyping panel was designed and optimised to evaluate mucosal invariant T cells (MAITs) and γδ T cells with activation marker expression on each subset. Gating strategy, cell subsets and markers analysed are shown. [file Image_4.jpeg]

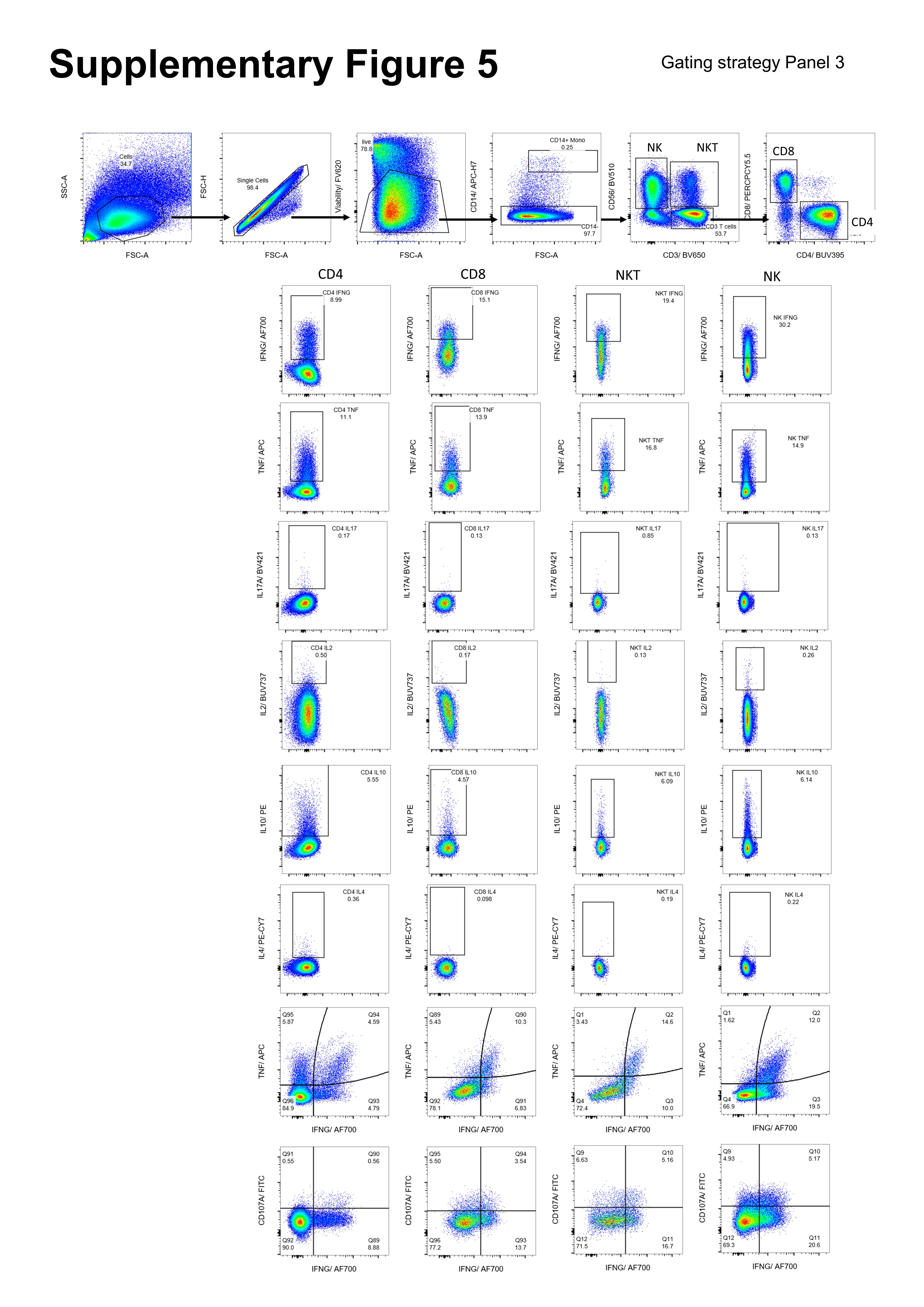

Supplement: Supplementary Figure 5 — Gating strategy for immunophenotyping and functionality analysis using panel 3. A 15-colour functionality panel was designed and optimised to evaluate six cytokines and a degranulation marker on multiple immune cell subsets. Gating strategy, cell subsets and functional markers are shown. [file Image_5.jpeg]

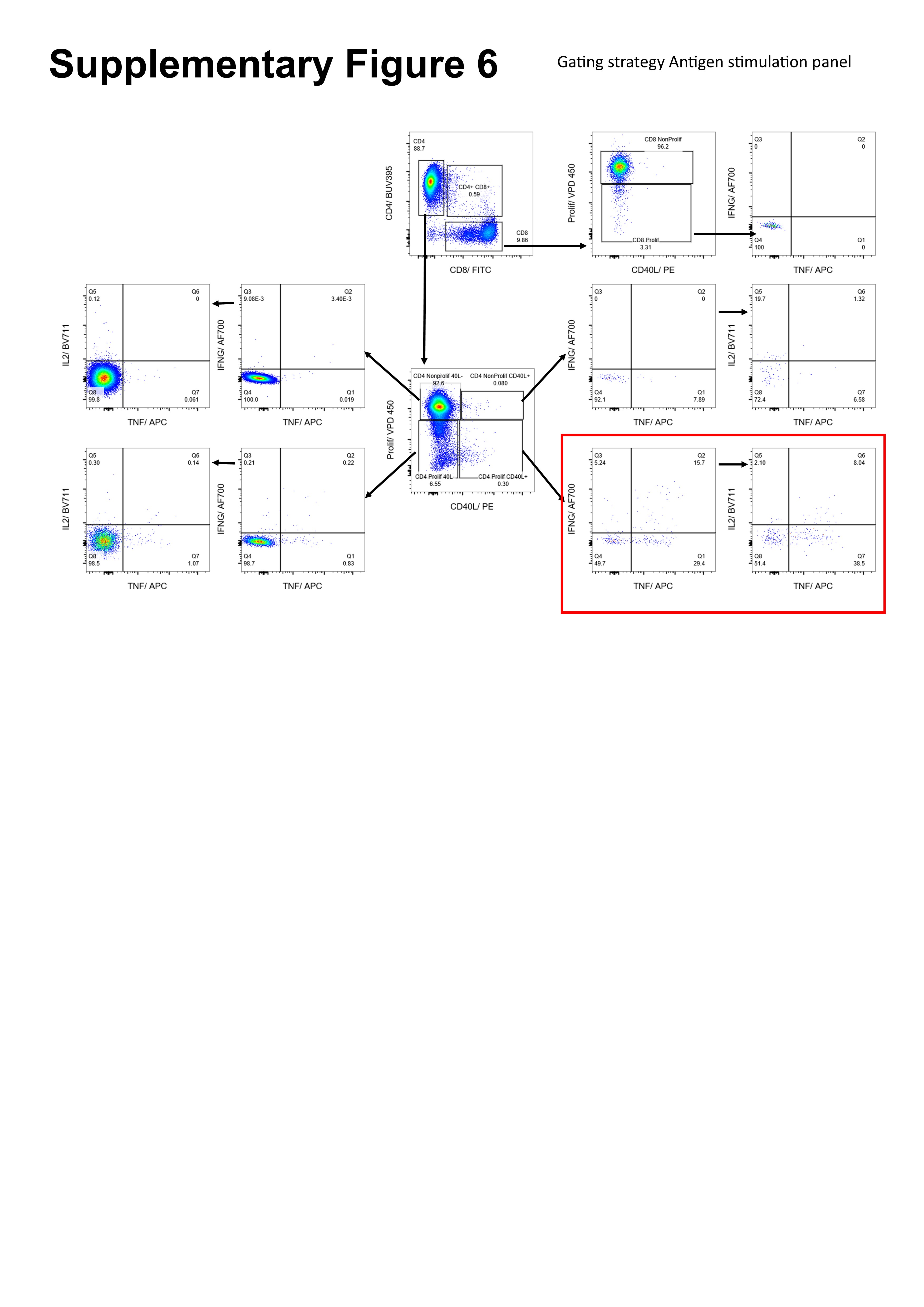

Supplement: Supplementary Figure 6 — Gating strategy for Antigen-specific proliferation and functionality testing panel 4. A 12-colour phenotyping and functionality testing panel that included proliferation and cytokine responses was designed and optimised. Gating strategy, cell subsets and functional markers are shown. [file Image_6.jpeg]
